# Disrupting the Cdk9/Cyclin T1 heterodimer of 7SK snRNP for the Brd4 and AFF1/4 guided reconstitution of active P-TEFb

**DOI:** 10.1093/nar/gkab1228

**Published:** 2021-12-22

**Authors:** Kai Zhou, Songkuan Zhuang, Fulong Liu, Yanheng Chen, You Li, Shihui Wang, Yuxuan Li, Huixin Wen, Xiaohua Lin, Jie Wang, Yue Huang, Cailing He, Nan Xu, Zongshu Li, Lang Xu, Zixuan Zhang, Lin-Feng Chen, Ruichuan Chen, Min Liu

**Affiliations:** State Key Laboratory of Cellular Stress Biology, School of Life Sciences, Xiamen University, Xiamen 361005, Fujian, China; State Key Laboratory of Cellular Stress Biology, School of Life Sciences, Xiamen University, Xiamen 361005, Fujian, China; State Key Laboratory of Cellular Stress Biology, School of Life Sciences, Xiamen University, Xiamen 361005, Fujian, China; Department of Biochemistry, University of Illinois at Urbana-Champaign, Urbana, IL 61801, USA; Biomolecular Interaction Centre, University of Canterbury, Christchurch 8140, New Zealand; State Key Laboratory of Cellular Stress Biology, School of Life Sciences, Xiamen University, Xiamen 361005, Fujian, China; State Key Laboratory of Cellular Stress Biology, School of Life Sciences, Xiamen University, Xiamen 361005, Fujian, China; State Key Laboratory of Cellular Stress Biology, School of Life Sciences, Xiamen University, Xiamen 361005, Fujian, China; State Key Laboratory of Cellular Stress Biology, School of Life Sciences, Xiamen University, Xiamen 361005, Fujian, China; State Key Laboratory of Cellular Stress Biology, School of Life Sciences, Xiamen University, Xiamen 361005, Fujian, China; State Key Laboratory of Cellular Stress Biology, School of Life Sciences, Xiamen University, Xiamen 361005, Fujian, China; State Key Laboratory of Cellular Stress Biology, School of Life Sciences, Xiamen University, Xiamen 361005, Fujian, China; State Key Laboratory of Cellular Stress Biology, School of Life Sciences, Xiamen University, Xiamen 361005, Fujian, China; State Key Laboratory of Cellular Stress Biology, School of Life Sciences, Xiamen University, Xiamen 361005, Fujian, China; State Key Laboratory of Cellular Stress Biology, School of Life Sciences, Xiamen University, Xiamen 361005, Fujian, China; State Key Laboratory of Cellular Stress Biology, School of Life Sciences, Xiamen University, Xiamen 361005, Fujian, China; Department of Biochemistry, University of Illinois at Urbana-Champaign, Urbana, IL 61801, USA; State Key Laboratory of Cellular Stress Biology, School of Life Sciences, Xiamen University, Xiamen 361005, Fujian, China; State Key Laboratory of Cellular Stress Biology, School of Life Sciences, Xiamen University, Xiamen 361005, Fujian, China

## Abstract

P-TEFb modulates RNA polymerase II elongation through alternative interaction with negative and positive regulation factors. While inactive P-TEFbs are mainly sequestered in the 7SK snRNP complex in a chromatin-free state, most of its active forms are in complex with its recruitment factors, Brd4 and SEC, in a chromatin-associated state. Thus, switching from inactive 7SK snRNP to active P-TEFb (Brd4/P-TEFb or SEC/P-TEFb) is essential for global gene expression. Although it has been shown that cellular signaling stimulates the disruption of 7SK snRNP, releasing dephosphorylated and catalytically inactive P-TEFb, little is known about how the inactive released P-TEFb is reactivated. Here, we show that the Cdk9/CycT1 heterodimer released from 7SK snRNP is completely dissociated into monomers in response to stress. Brd4 or SEC then recruits monomerized Cdk9 and CycT1 to reassemble the core P-TEFb. Meanwhile, the binding of monomeric dephosphorylated Cdk9 to either Brd4 or SEC induces the autophosphorylation of T186 of Cdk9. Finally, the same mechanism is employed during nocodazole released entry into early G1 phase of cell cycle. Therefore, our studies demonstrate a novel mechanism by which Cdk9 and CycT1 monomers are reassembled on chromatin to form active P-TEFb by its interaction with Brd4 or SEC to regulate transcription.

## INTRODUCTION

In eukaryotic cells, RNA polymerase II (Pol II) is strictly regulated by several tightly coordinated steps in response to transcriptional demands. Transcription initiation and elongation are the two key rate-limiting steps that control gene expression. While much is known about the regulation in transcription initiation, the regulatory mechanisms governing elongation have yet to be fully understood (1,2). During transcription elongation, modulation of promoter proximal pause and release of Pol II is crucial for the rapid expression of many inducible genes that play key roles in growth, differentiation, and stress response (3–5).

Currently, multiple positive and negative factors that directly target Pol II have been identified as key regulators in transcription elongation, including the positive factor Positive Transcription Elongation Factor b (P-TEFb), the negative factor DRB sensitivity-inducing factor (DSIF), and negative elongation factor (NELF) (3,6). Shortly after transcribing the first 20–120 nucleotides, Pol II is stalled by DSIF and NELF, resulting in pausing at the promoter-proximal region and temporarily halting the transcription of >30% of active genes (7,8). Upon stimulation, multiple P-TEFbs are quickly recruited to the stalled Pol II, where they phosphorylate DSIF and NELF to release Pol II (1,2). Phosphorylation of Ser2 at the C-terminal domain (CTD) of Pol II by P-TEFb also allows productive elongation to produce full-length mRNA (1,2). Abnormal regulation of transcription elongation has been linked to cancer and other human diseases, highlighting the importance of P-TEFb-mediated elongation control (1,9).

The core P-TEFb, which consists of Cdk9 and CycT1, is a general transcription factor which can bind to different complexes to modulate the appropriate transcriptional activity (2,10). Most core P-TEFbs in the cells exist in a chromatin-free, inactive 7SK snRNP complex, which is comprised of the 7SK snRNA (7SK) and three nuclear proteins HEXIM1 (or the minor HEXIM2), MePCE and Larp7 (11–15). Within the 7SK snRNP complex, 7SK serves as the central scaffold to maintain the integrity of 7SK snRNP through interactions with its protein components. HEXIM1 functions as a kinase inhibitor of Cdk9 in a 7SK-dependent manner, while MePCE and LARP7 together stabilize 7SK (15). Another major portion of core P-TEFb is found to bind to recruitment factors Brd4 or the super elongation complex (SEC) in the active form, which in turn recruits P-TEFb onto the chromatin to facilitate the release of paused Pol II (16,17). Brd4 is a bromodomain containing protein which recruits P-TEFb to DSIF for subsequent phosphorylation (18). SEC is a multisubunit complex that contains one of four AFF scaffold proteins (AFF1 to AFF4), one of three ELL proteins (ELL1 to ELL3), and an ENL or its analogue AF9 (17,19). P-TEFbs recruited by SEC phosphorylate NELF and Ser2 on the CTD of Pol II (18,19). Thus, the cooperation between Brd4/P-TEFb and SEC/P-TEFb abolishes the inhibition of stalled Pol II, leading to the activation of global gene transcription (18). Notably, P-TEFb was previously considered as a cell cycle progression unrelated kinase, which is unique in the cyclin-dependent kinase (CDK) superfamily (20). The only link between P-TEFb and the cell cycle progression is P-TEFb's recruitment by Brd4 to target the early G1 genes (21–23).

When stimulated, cellular signaling triggers a functional switch of P-TEFb from its inactive 7SK snRNP form to the active Brd4/P-TEFb or SEC/P-TEFb forms (2,10). Our previous studies revealed that the core P-TEFb was released from 7SK snRNP by dephosphorylation of Cdk9 at threonine 186 (T186) through the cooperation of PP2B and PP1α (24). This process directly results in the dephosphorylated P-TEFb without full transcriptional activity, what remains unclear is how P-TEFb is reactivated after release from 7SK snRNP. In this study, we found that upon releasing from 7SK snRNP, the Cdk9/CycT1 heterodimer of core P-TEFb dissociates into monomers. The monomeric Cdk9 and CycT1 are then reassembled into active P-TEFb facilitated by Brd4 and SEC scaffold protein AFF1/AFF4. The functional dissociation and reconstitution of P-TEFb are essential for gene expression under stress conditions and during cell cycle progression.

## MATERIALS AND METHODS

### Materials

More detailed information about chemicals, antibodies and plasmids used in the study is provided in the Supplementary Information.

### Cell lines, transfection, infection and treatment with UV or pharmacological compounds

293T, HeLa and the HeLa-based F1C2 (Cdk9-f) cells were maintained as previously described (25). HCT116 cells were maintained in McCoy's 5A medium (Sigma) supplemented with 10% FBS and 1xPSK (Gibco). A549, NT2 and HepG2 cells were maintained in RPMI 1640 medium supplemented with 10% FBS and 1xPSK (Gibco). The transfection and lentiviral infection were performed as previously described (24,25). For pharmacological compounds treatment, cells at ∼50% confluence were pre-incubated with DMSO, Flavopiridol (FVP, 300 nM) or OA (200 nM) for 1 h, followed by the treatment with 10 mM HMBA for 2 h or other compounds at the indicated concentrations for 1 h. For UV irradiation, the cell medium was briefly removed during UV exposure at 80 J/m^2^ in Spectrolinker XL-1000 (Spectronics). Cells were then collected 1 h after UV irradiation for further analysis (19).

### Cell synchronization and mitotic shake-off

To obtain the population of mitotic cells, 80% confluence HeLa or F1C2 cells were placed in fresh medium containing nocodazole (50 ng/ml). After incubation for 24 h, the rounded-up mitotic cells were shaken off the culture dish, washed with drug-free medium twice, and then replated in fresh medium. Cells were harvested and extracted at different time points following the start of replating.

### Preparation of LSF, HSF and WCE

The low-salt fraction (LSF) and high-salt fraction (HSF) were prepared with improved nuclear fractionation protocol (see Supplementary Information for details). The whole cell extraction (WCE) was prepared as previously described (24,26).

### Immunoprecipitation and Western blot analysis

Immunoprecipitation (IP) and Western blot (WB) analysis were performed as previously described (18). Briefly, Flag-, HA-tagged proteins or endogenous proteins and their associated factors were isolated by anti-Flag, anti-HA or specific antibodies from LSF, HSF or WCE of transfected or lentivirus-infected HeLa cells as previously described (18). The levels of proteins in IP products, fractionated samples or whole cell extracts were analyzed by WB with corresponding antibodies.

### *In vitro* phosphatase treatment of affinity-purified P-TEFb

The *in vitro* phosphatase treatment were performed as previously described (24). Briefly, the 7SK snRNP or active P-TEFb was affinity-purified from 300 μL of F1C2 LSF or HSF, respectively. After washing with buffer D (20 mM HEPES–KOH PH 7.9, 0.1% NP40, 10% glycerol, 0.2 mM EDTA) containing 0.3 M KCl (designated as D0.3) and then 0.1 M KCl (designated as D0.1), the immobilized complex on anti-Flag beads was incubated for 30 min at 30°C with the indicated amounts of phosphatases in the 30 μl of *in vitro* assay buffer (10 mM HEPES–KOH PH 7.9, 10 mM KCl) plus 1 mM MnCl_2_ and CaCl_2_. The reactions were carried in the presence or absence of 1 μg/μl RNase as indicated. The levels of pT186 and other associated proteins were detected by WB.

### *In vitro* kinase assay

For Cdk9 autophosphorylation assay, the dephosphorylated Cdk9 monomers were affinity-purified from LSF of HMBA-treated F1C2 cells. Wild-type Brd4, SEC components and their truncations were affinity-purified from WCE of transiently transfected 293T cells after HMBA treatment. To ensure the high purity of indicated proteins, the NaCl concentration of LSF or WCE was adjusted to 0.8 M and then pre-depleted with anti-CycT1 or anti-Cdk9 antibody, respectively. The *in vitro* kinase assay was performed as previously described (18). The levels of pT186 were determined by WB with anti-phospho-T186 antibody.

### *In vitro* pull-down assay

For *in vitro* reconstitution of core P-TEFb, immobilized CycT1, Brd4, AFF1 or AFF4 were derived from WCE of transfected 293T cells by anti-HA IP, washed 3 times with D0.3 and then once with D0.1. After incubation for 30 min at 30°C with the indicated proteins in 30 μl *in vitro* assay buffer, samples were washed, and finally eluted with HA peptides. The levels of the desired proteins were detected by WB.

### Quantitative RT-PCR analyses

The quantitative real-time polymerase chain reaction (qRT-PCR) was performed as previously described (18). The primer sequences are shown in the Supplementary Information. All values were expressed as mean ± SD of three replicates. *P*-values were assessed using two-tailed Student's *t*-test.

### Luciferase assay

HeLa cells with an integrated HIV-LTR-luciferase reporter gene (HIV-LTR-Luc) were incubated with 10 mM of HMBA and 100 μg/ml synthesized peptides for 6 h as indicated. The luciferase activity was measured as previously described (18). Data from three replicates were averaged and presented as fold induction compared to untreated cells. All values were expressed as Mean ± SD of three replicates. *P*-values were assessed using two-tailed Student's *t*-test.

### Chromatin immunoprecipitation (ChIP)-qPCR

ChIP was performed in HeLa cells and the immunoprecipitated DNA was analyzed by qRT-PCR as previously described (26). All values were expressed as Mean ± SD of three replicates. *P*-values were assessed using two-tailed Student's *t*-test. The primer sequence are presented in Supplementary Information.

## RESULTS

### Stress induces the core P-TEFb of 7SK snRNP to completely dissociate into Cdk9 and CycT1 monomers

**Figure 1.**
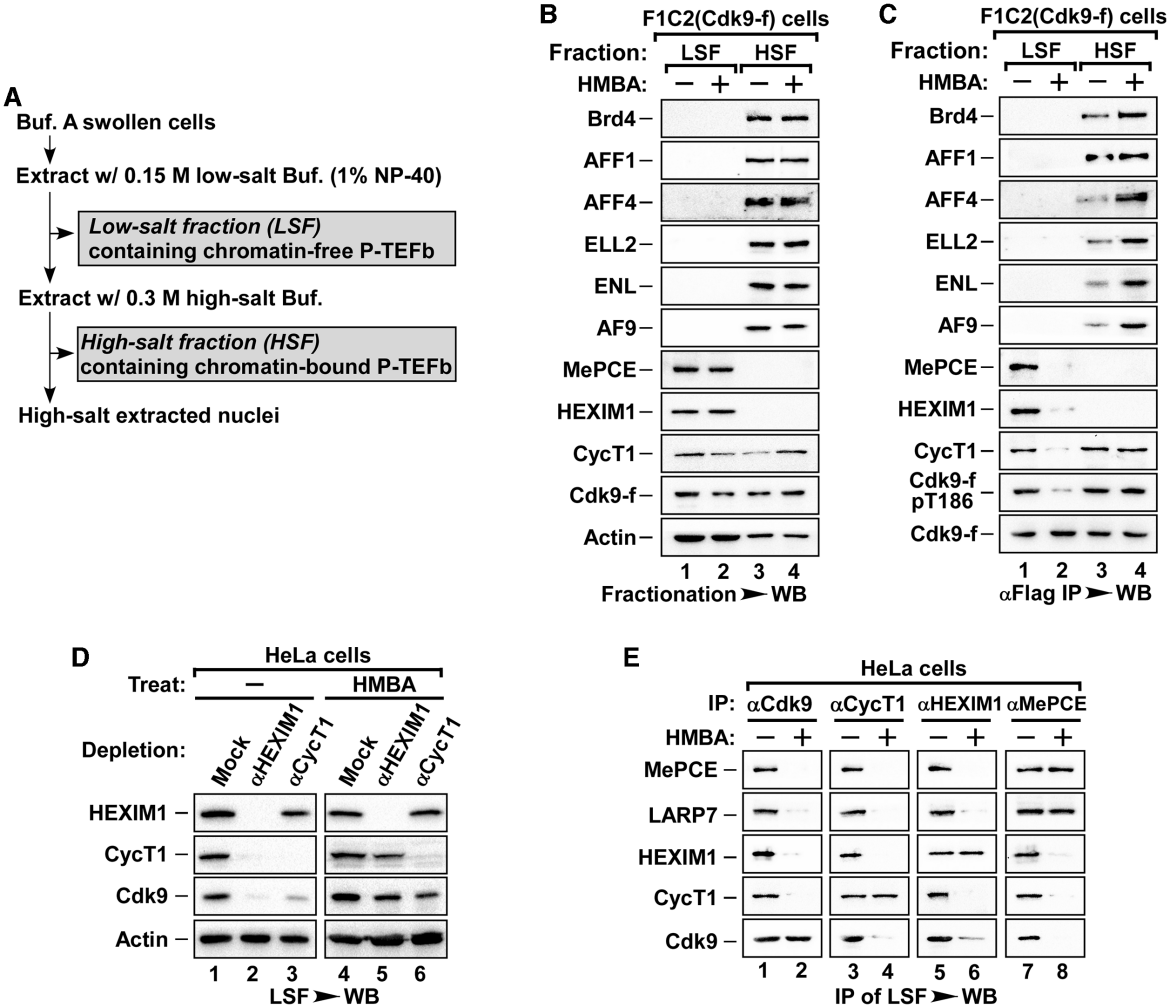
The Cdk9/CycT1 heterodimer of chromatin-free 7SK-snRNP is dissociated into monomers upon stimulation. (**A**) Schematic representation of an improved nuclear fractionation protocol. (**B**) The low-salt fraction (LSF) and high-salt fraction (HSF) prepared from F1C2 (Cdk9-f stable line) cells treated with or without HMBA were subjected to WB and probed with the indicated antibodies. (**C**) The Cdk9 associated proteins and T186 phosphorylation in anti-Flag immunoprecipitates (IPs) from LSF and HSF of F1C2 cells treated with or without HMBA were analyzed by WB. (**D**) LSF of HeLa cells was either mock- or immuno-depleted with immobilized anti-HEXIM1 or anti-CycT1 antibodies before or after HMBA treatment. The levels of Cdk9, CycT1, HEXIM1 and Actin (internal control) in the depleted LSF were examined by WB. (**E**) HeLa LSF with or without HMBA treatment was subjected to endogenous IP with the indicated specific antibodies and blotted for their associated proteins by WB.

To study the process of P-TEFb activation, we established a cell fractionation method to separate P-TEFbs based on their state of transcriptional activity. To accomplish this, we fractionated F1C2 cells, a HeLa cell line stably expressing Flag-tagged Cdk9 (Cdk9-f), into two fractions, the low-salt fraction (LSF) and the high salt fraction (HSF) (Figure 1A). The LSF extracted with 0.15 M NaCl low salt buffer was comprised of cytoplasmic and nucleoplasmic extracts with the inactive P-TEFb in complex with 7SK-snRNP (Figure 1B, lane 1). The HSF was extracted with 0.3M NaCl high salt buffer of the remaining nuclei, which was comprised of chromatin extracts with active P-TEFb in complex with Brd4 and SEC (Figure 1B, lane 3). This distribution of both inactive and active P-TEFb complexes remained separate when cells were treated with hexamethylene bisacetamide (HMBA), a drug capable of inducing the release of P-TEFb from 7SK snRNP and the subsequent recruitment by Brd4 or SEC (Figure 1B, lanes 2 and 4). Immunoprecipitation of Cdk9-f in LSF and HSF confirmed the interacting components within inactive or active P-TEFb complexes after fractionation (Figure 1C). P-TEFb was released from 7SK snRNP and was recruited by Brd4 and SEC after HMBA treatment (Figure 1C).

Surprisingly, in the LSF of HMBA-treated F1C2 and HeLa cells, in addition to the disruption of 7SK snRNP, we observed a dissociation of Cdk9/CyclinT1 heterodimer, the core P-TEFb (Figure 1C, lane 2 and Supplementary Figure S1A, right panel). We explored whether this observation was limited to HMBA treatment or HeLa cell line. Different physical and chemical stress capable of activating P-TEFb all caused a similar dissociation of Cdk9 and CycT1 (Supplementary Figure S1B). Similarly, HMBA treatment also caused core P-TEFb dissociation in the LSF in multiple cell lines (Supplementary Figure S1C). To further confirm the dissociation, we performed a depletion assay targeting HEXIM1 or CycT1 in LSF of HeLa cells. Compared to untreated cells, depletion of HEXIM1 failed to co-deplete Cdk9 or CycT1 in HMBA-treated cells, likely due to the induced release of P-TEFb from 7SK snRNP (Figure 1D, lanes 2 and 5). Importantly, CycT1 could not co-deplete Cdk9 in HMBA-treated cells (Figure 1D, lanes 3 and 6), confirming the core P-TEFb was indeed dissociated during HMBA-induced activation. In fact, when different factors of 7SK snRNP were immunoprecipitated after HMBA treatment, we found that the only remaining interaction of the disrupted 7SK snRNP was with MePCE and LARP7 (Figure 1E).

### PP1α-mediated Cdk9-pT186 dephosphorylation leads to core P-TEFb dissociation

We have previously reported that phosphorylated T186 (pT186) of Cdk9 is directly dephosphorylated by PP1α when induced by HMBA or UV, leading to the disruption of the 7SK snRNP (24). Given the same dephosphorylation was observed in T186 along with the dissociation of CycT1 (Figure 1C, lane 2), we asked whether pT186 dephosphorylation by PP1α was also important for core P-TEFb dissociation in the LSF.

**Figure 2.**
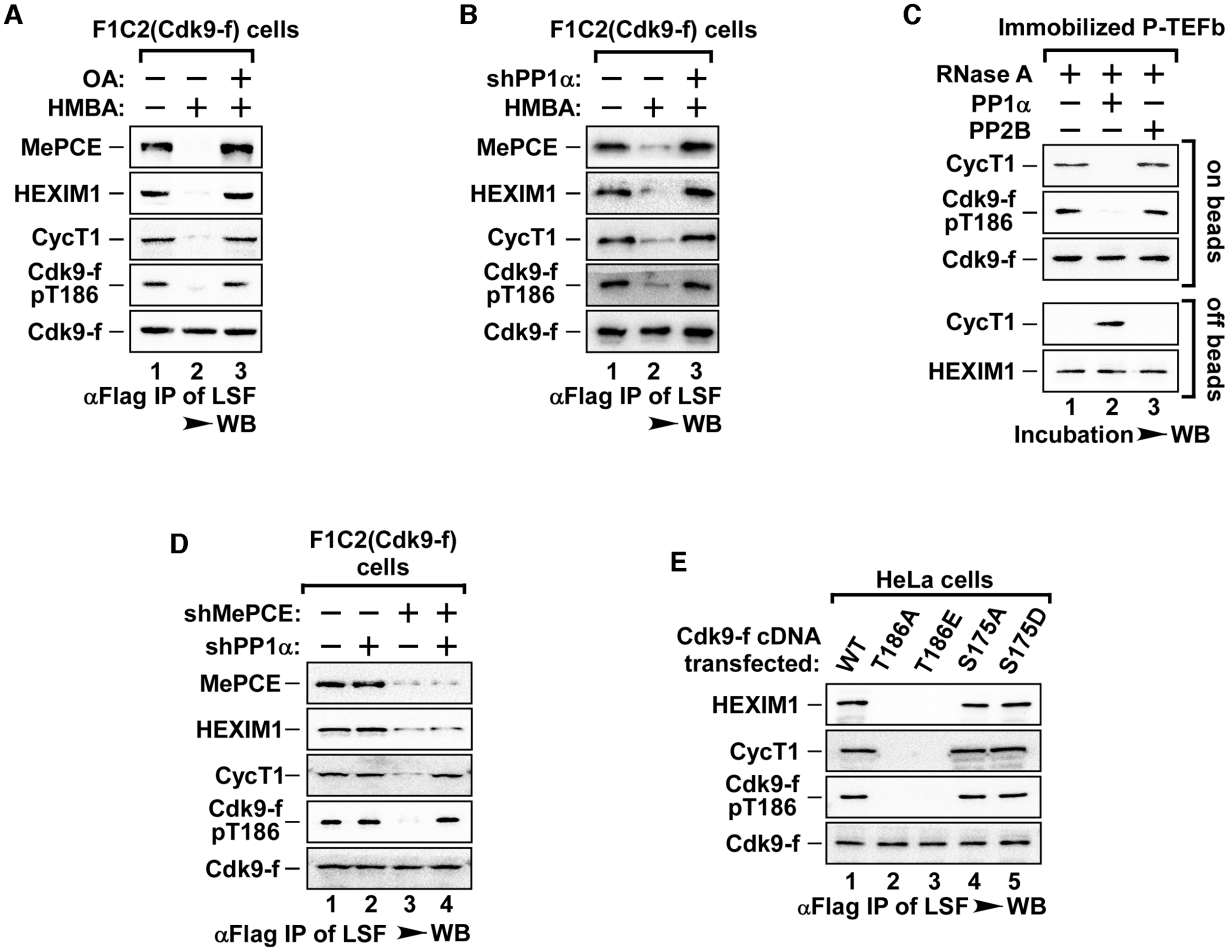
The dephosphorylation of Cdk9 at T186 by PP1α triggered the dissociation of core P-TEFb. (**A**) F1C2 cells were preincubated with PP1α inhibitor Okadaic Acid (OA) and then treated with HMBA. Anti-Flag IPs from LSF were subjected to WB for the levels of pT186 and Cdk9-f-bound proteins. (**B**) F1C2 cells were infected with shPP1α and then stimulated by HMBA. Anti-Flag IPs from LSF were analyzed as in A. (**C**) Affinity-purified P-TEFb from LSF of F1C2 cells was first incubated with RNase A, and then with the indicated PP2B (2 U) or PP1α (2 U). The reaction samples were analyzed as in B. (**D**) LSF derived from F1C2 cells infected with the indicated shRNA(s) was subjected to anti-Flag IP and then analyzed by WB to determine the levels of phosphorylated T186 (pT186) and the indicated Cdk9-f-associated proteins. (**E**) HeLa cells were transfected with various mutants of Cdk9-f. Anti-Flag IPs from LSF were subjected to WB for the levels of pT186 and the indicated Cdk9-f-associated factors.

Consistent with our previous study, when cells were pre-treated with Okadaic Acid (OA), a PP1α inhibitor, HMBA treatment failed to disrupt 7SK snRNP (Figure 2A). Dissociation of core P-TEFb was also blocked, likely due to the inhibition of PP1α-mediated pT186 dephosphorylation (Figure 2A). In support of this notion, shRNA depletion of PP1α also blocked HMBA-induced core P-TEFb dissociation (Figure 2B). However, these data did not exclude the possibility that core P-TEFb was unable to dissociate when in complex with the inhibitory 7SK subcomplex (7SK, HEXIM1, MePCE and Larp7). The levels of the proteins tested remained unchanged with OA treatment and shRNA knockdown (Supplementary Figures S2A and S2B).

To determine whether dephosphorylation of pT186 by PP1α was the direct cause of core P-TEFb dissociation, we first took an *in vitro* approach by immobilizing inactive P-TEFb complex isolated from the LSF of F1C2 cells with Flag beads, RNase A was then added to degrade 7SK snRNA and release the inhibitory 7SK subcomplex off beads (Figure 2C and Supplementary Figure S2C), leaving only the core P-TEFb immobilized on-beads. When PP1α was added, pT186 was dephosphorylated and Cdk9/CycT1 heterodimer dissociated (Figure 2C, lane 2). This dissociation was not observed with the addition of PP2B, a protein phosphatase without the ability to dephosphorylate pT186 (24).

To complement the above experiment, we depleted MePCE, a key subunit required for the stability of inhibitory 7SK subcomplex, in F1C2 cells using shRNA (Supplementary Figure S2D). Without the sequestration of P-TEFb by 7SK snRNP, Cdk9-pT186 was dephosphorylated and the core P-TEFb dissociated (Figure 2D, lane 3). In contrast, when PP1α was also knocked down, T186 remained phosphorylated with the core P-TEFb remaining intact despite the release of 7SK subcomplex (Figure 2D, lane 4). Furthermore, Cdk9-T186A/E phosphorylation-null mutant failed to pull down CycT1 compared to WT Cdk9 or other phosphorylation mutants (Figure 2E). The levels of proteins tested remained unchanged with overexpressed Cdk9 mutants (Supplementary Figure S2E). Collectively, these data implicate that PP1α-mediated dephosphorylation of pT186 is indeed the direct cause of dissociation of core P-TEFb. Notably, the *in vivo* dephosphorylation of pT186 occurred without HMBA-induced activation of P-TEFb (Figure 2D), suggesting that PP1α is constitutively active in the cells, and the function of the inhibitory 7SK subcomplex is likely to protect pT186 from dephosphorylation, thus keeping core P-TEFb intact.

### The presence of Brd4 and AFF1/AFF4 is essential for the reassembly of core P-TEFb

**Figure 3.**
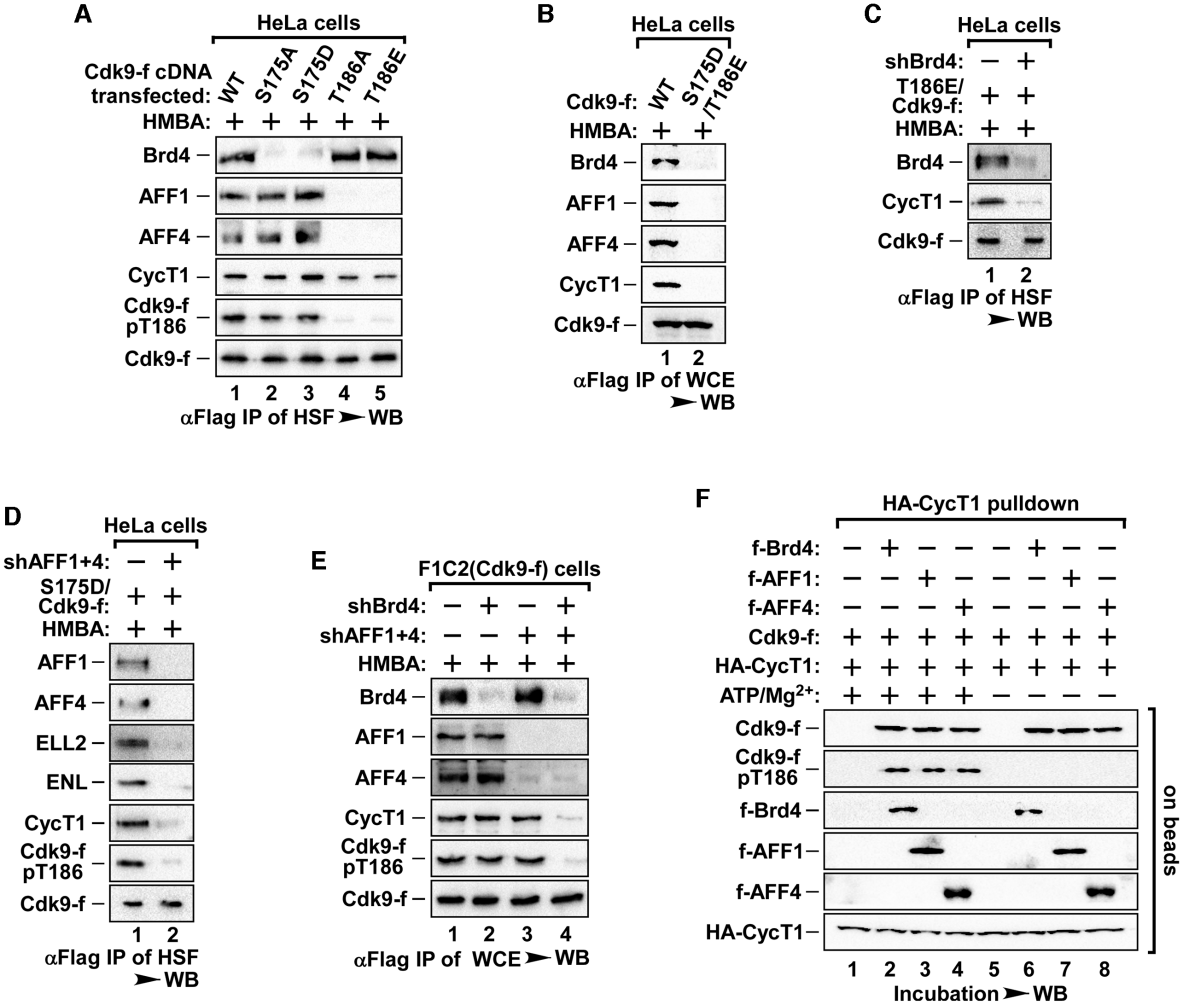
Brd4 and SEC are responsible for core P-TEFb reassembly from dissociated monomers. (**A**, **B**) IPs derived from HSF or WCE of HeLa cells expressing WT or mutant Cdk9-f were analyzed by WB as indicated. All cells in (A) to (E) were treated with HMBA before harvesting. (**C**) IPs derived from HSF of HeLa cells co-transfected with T186E-Cdk9-f cDNA and indicated shRNAs were analyzed by WB. (**D**) IPs derived from HSF of HeLa cells with S175D-Cdk9-f cDNA and indicated shRNA co-transfection were analyzed by WB. (**E**) IPs derived from WCE of F1C2 cells infected with the indicated shRNAs were analyzed by WB. (**F**) The *in vitro* pull-down assay for the binding of Cdk9-f to immobilized HA-CycT1 was performed in the presence of Brd4, AFF1 or AFF4 with/without ATP/Mg^2+^. The levels of Cdk9-f bound to HA-CycT1 and pT186 were analyzed by WB.

Cdk9-pT186 dephosphorylation led to the dissociation of core P-TEFb from 7SK snRNP into Cdk9 and CycT1 monomers (Figures 1 and 2), raising a possibility that the reconstitution of active P-TEFb might require both T186 re-phosphorylation and reassembly of core P-TEFb from monomers. To test this possibility, we first determined whether T186 re-phosphorylation would drive reassembly of P-TEFb. We expressed Flag-tagged WT-Cdk9, Cdk9-T186A/E mutant or Cdk9-S175A/D mutant to similar levels in HeLa cells and examined their reassembly with CycT1 in the HSF, the fraction containing active P-TEFb. Unlike their interactions in the LSF (Figure 2E), Cdk9-T186A or Cdk9-T186E mutant was able to bind to CycT1, similar to WT-Cdk9 and S175 mutants upon HMBA treatment (Figure 3A). These data suggest that T186 re-phosphorylation might be critical for the kinase function of active P-TEFb, it may not be a prerequisite for the reassembly of core P-TEFb. Interestingly, we observed that Cdk9-S175 and Cdk9-T186 mutants exclusively bound to SEC or Brd4, respectively (Figure 3A, lanes 2–5), while the ectopic expression of Cdk9 mutants did not affect the levels of either Brd4 or SEC in the HSF (Supplementary Figure S3A).

Given Brd4 and SEC are the major chromatin recruitment factors for active P-TEFb (16–18), this result prompted us to test whether they might also play a role in core P-TEFb reassembly. Supporting this notion, we found that the Cdk9-S175D/T186E dual mutant which abolished its interaction with both Brd4 and SEC, failed to reassemble with CycT1 in HeLa cells, as compared to WT-Cdk9 (Figure 3B and Supplementary Figure S3B). To further explore individual role of Brd4 and SEC in core P-TEFb reconstitution, we depleted Brd4 or SEC with shRNAs in HeLa cells expressing Cdk9 T186E and S175D. When Brd4 was depleted, Cdk9-T186E failed to reassemble with CycT1 (Figure 3C). Moreover, when we co-depleted three paralog components of SEC (AFF1/4, ELL1/2, AF9 + ENL) in Cdk9-S175D expressing HeLa cells, the co-depletion of SEC scaffold proteins AFF1 + 4, but not ELL1/2 or AF9 + ENL, blocked core P-TEFb formation after HMBA treatment (Figure 3D and Supplementary Figure S3C). The level of CycT1, as well as the levels of non shRNA targeted SEC components in the HSF remain unchanged with shRNA-knockdown of Brd4 or SEC (Supplementary Figures S3D and S3E).

Having demonstrated that Brd4 and AFF1/4 are involved in the formation of core P-TEFb with Cdk9 T186E and S175D mutants, we next evaluated whether Brd4 or SEC also affected core P-TEFb reassembly with WT-Cdk9. When we depleted Brd4 or AFF1 + 4 individually in F1C2 cells, we found that HMBA failed to disrupt the formation of core P-TEFb between WT-Cdk9 and CycT1 (Figure 3E). The disruption of core P-TEFb formation only occurred with the co-depletion of both Brd4 and AFF1 + 4 (Figure 3E and Supplementary Figure S3F). These data indicate that Brd4 and SEC have redundant functions in facilitating core P-TEFb reassembly.

To verify the above observations, we performed *in vitro* pull-down assay with purified HA-CycT1 monomers immobilized on-beads. When incubated with affinity-purified, dephosphorylated Cdk9-f monomers, HA-CycT1 failed to pull down Cdk9 (Figure 3F, lane 1). With the addition of Brd4, Cdk9 and CycT1 were able to form the core P-TEFb, accompanied by the re-phosphorylation of T186 in the presence of ATP/Mg^2+^ (Figure 3F, lane 2). Without ATP/Mg^2+^ to facilitate re-phosphorylation, reassembly of core P-TEFb still occurred upon Brd4 co-incubation, consistent with the previous conclusion that T186 phosphorylation state does not affect reassembly of core P-TEFb (Figure 3A). Similar results were obtained with AFF1 (Figure 3F, lanes 3 and 7) and AFF4 (Figure 3F, lanes 4 and 8). Together, our data reveal that both Brd4 and AFF1/4 facilitate the reassembly of core P-TEFb, a key step towards active P-TEFb reconstitution.

### Brd4 and SEC are required for the autophosphorylation of T186 of Cdk9 to restore activity

**Figure 4.**
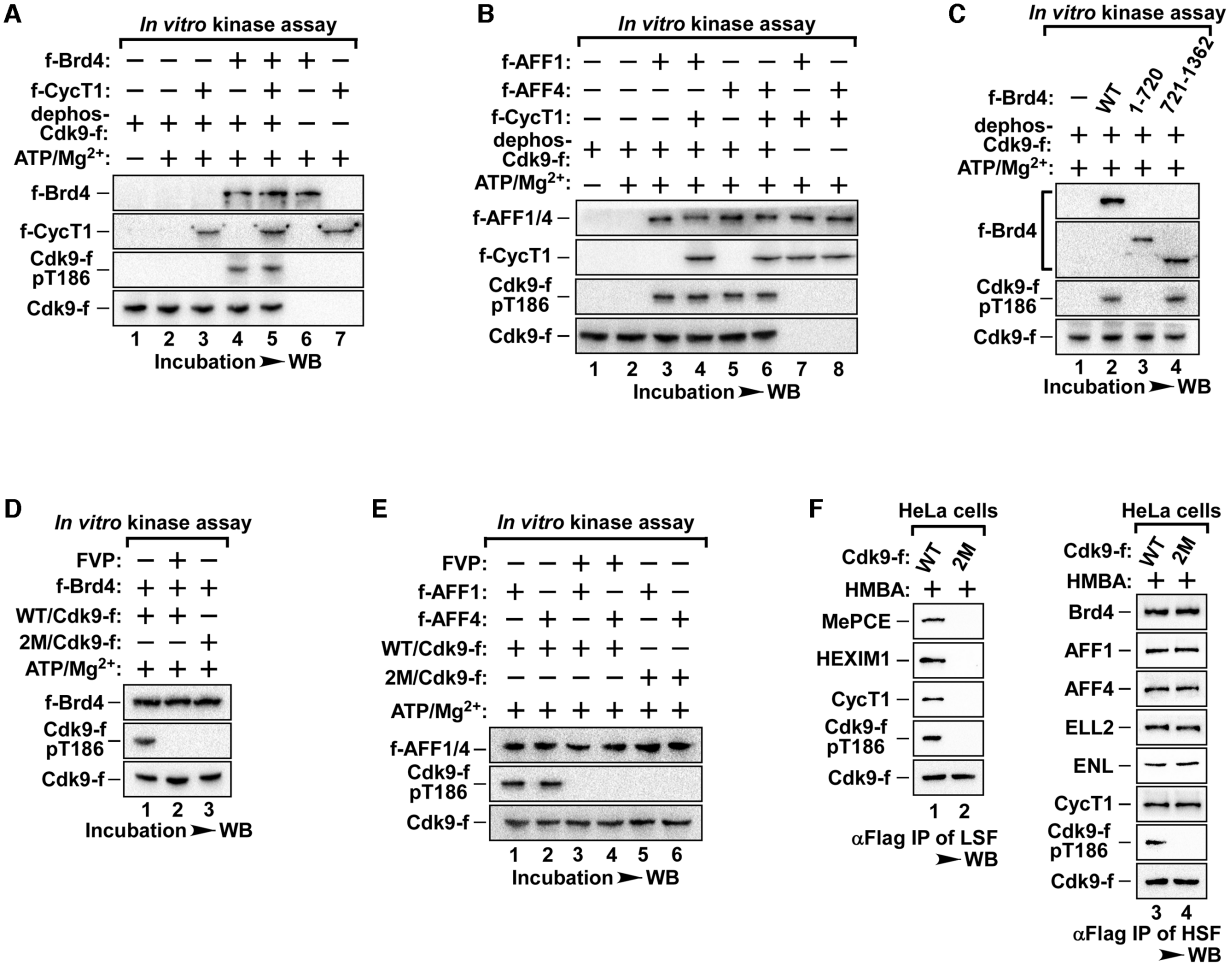
Brd4 and AFF1/4 induced autophosphorylation of Cdk9-T186. (**A**, **B**) The *in vitro* kinase assay was performed with purified f-Brd4, f-AFF1, f-AFF4, f-CycT1 and dephosphorylated Cdk9-f as indicated and the pT186 of Cdk9-f was detected by WB with specific anti-phospho-T186 Cdk9 antibody. (**C**) Levels of pT186 in kinase reactions containing affinity purified dephosphorylated Cdk9-f and full-length or truncated f-Brd4 were analyzed by WB as indicated. (**D**, **E**) Kinase reactions containing purified f-Brd4, f-AFF1, f-AFF4, WT- or 2M-Cdk9-f with or without FVP are shown as indicated. The levels of pT186 was examined as in B. 2M-Cdk9 is a double-point mutant of Cdk9 (K48R/E66A). (**F**) HeLa cells were transfected with WT- or 2M-Cdk9-f. Anti-Flag IPs from LSF or HSF were analyzed by WB for the levels of pT186 and the indicated Cdk9-f-associated factors.

Next, we wanted to understand how Cdk9-T186 is re-phosphorylated during the reconstitution of active P-TEFb. Given that with the addition of Brd4 or AFF1/4, Cdk9 and CycT1 was sufficient to trigger the phosphorylation of T186 (Figure 3F), we asked which of these factors were sufficient to facilitate T186 re-phosphorylation. To determine this, we performed an *in vitro* kinase assay. When dephosphorylated Cdk9 monomers were incubated with ATP/Mg^2+^, no T186 phosphorylation could be detected (Figure 4A, lane 2). The addition of CycT1 also failed to trigger T186 re-phosphorylation (Figure 4A, lane 3). However, T186 was phosphorylated when Brd4 was added with Cdk9 (Figure 4A, lane 4) and this phosphorylation was enhanced with the addition of Brd4 in a dose-dependent-manner (Supplementary Figure S4A) while no enhanced phosphorylation was observed when CycT1 monomers were added (Figure 4A, lane 5). Similar results were obtained with AFF1/4 under the same conditions (Figure 4B). Since Brd4 has previously been reported as an atypical kinase with its kinase activity mapped within the N-terminus (27), we tested whether this kinase activity was responsible for T186 re-phosphorylation. To our surprise, only Brd4 C-terminal mutant (721–1362) but not the N-terminal mutant (1–720) was able to facilitate T186 phosphorylation (Figure 4C). Additionally, N-terminus of both AFF1 (1–328) and AFF4 (1–300) also induced T186 re-phosphorylation (Supplementary Figure S4B). Interestingly, these Brd4 or AFF mutants with the ability to trigger the re-phosphorylation of Cdk9 were also able to facilitate the reassembly of core P-TEFb (Supplementary Figures S4C and S4D).

Since neither the C-terminus of Brd4, nor AFF1/4 contains any known kinase domains, the data above suggest that Cdk9 may undergo autophosphorylation of T186 induced by Brd4 or AFF1/4. To explore this possibility, we generated a kinase-dead Cdk9-f (KD-Cdk9) with a double point mutation in the conserved catalytic pocket (K48R/E66A). A pharmacological approach was also taken with flavopiridol (FVP), a potent inhibitor that competes with ATP to inhibit CDKs. In contrast to the stimulatory effect from Brd4 or AFF1/4, both FVP and the KD-Cdk9 inhibited the re-phosphorylation of Cdk9 in an *in vitro* kinase assay (Figures 4D and 4E). Furthermore, Cdk9 remained unphosphorylated at T186 in either the LSF or HSF when HeLa cells were transfected with either WT or KD-Cdk9, while their interactions with Brd4 or SEC in the HSF remained undisrupted (Figure 4F). Collectively, these data implicate that Brd4 and AFF1/4 are the key factors for the re-phosphorylation of Cdk9-T186, likely by its autophosphorylation.

### The direct interaction between Brd4/AFF1/AFF4 and Cdk9 T-loop is key for the reconstitution of active P-TEFb

**Figure 5.**
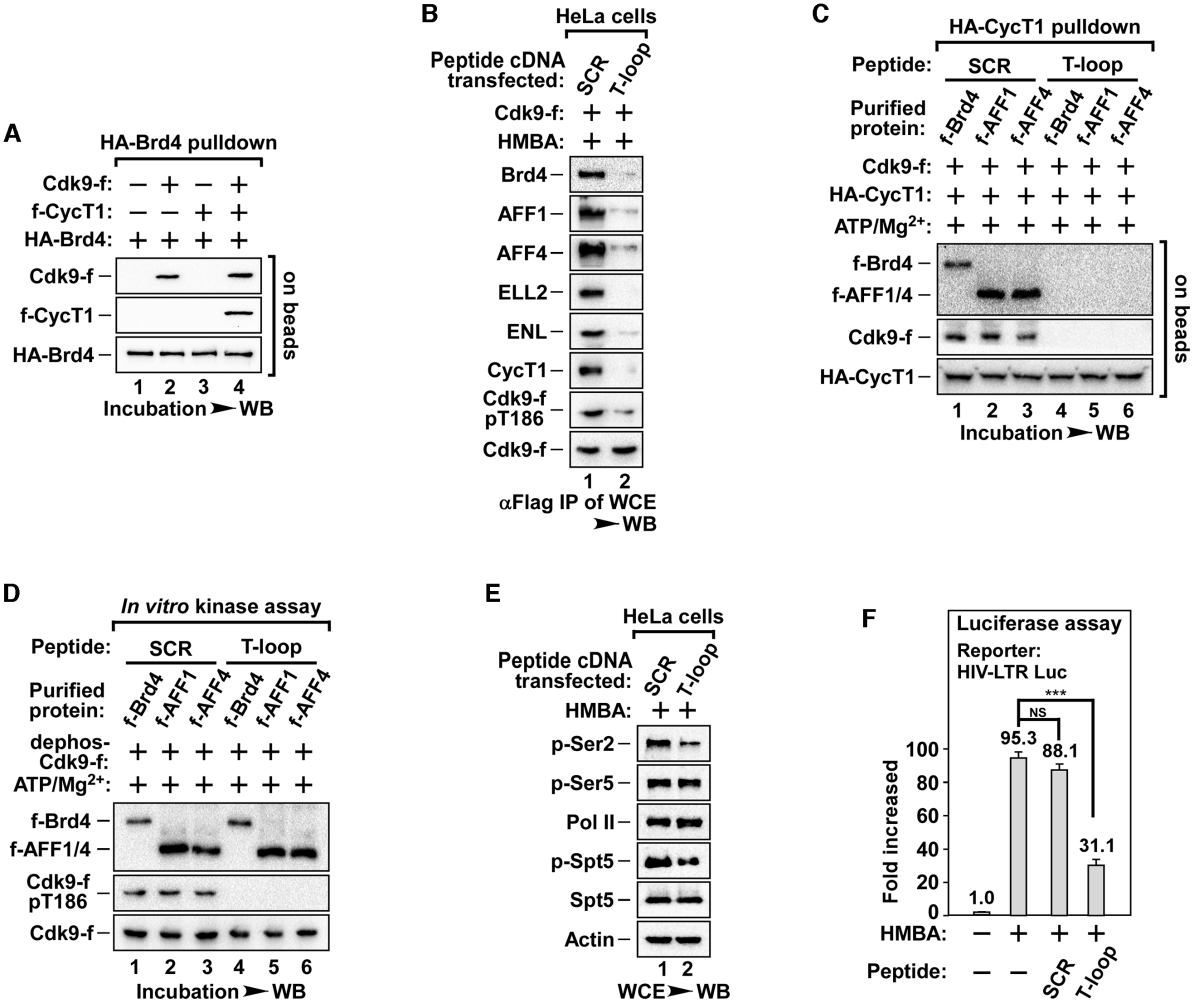
The T-loop peptide of Cdk9 blocks Brd4/AFF1/AFF4 induced reconstitution of active P-TEFb and thus impairs transcription. (**A**) Pull-down reactions containing affinity purified HA-Brd4, Cdk9-f and f-CycT1 are shown as indicated. The amounts of Cdk9-f and f-CycT1 bound to the immobilized HA-Brd4 were analyzed by WB. (**B**) The anti-Flag IPs derived from WCE of HeLa cells co-transfected with Cdk9-f cDNA and SCR (as control) or T-loop peptide under HMBA treatment were analyzed by WB for the levels of pT186 and Cdk9-f-bound proteins. (**C**) The *in vitro* pull-down reactions containing the indicated purified proteins and 100 μg/mL SCR or T-loop peptide are shown as indicated. The amounts of Cdk9-f bound to the immobilized HA-CycT1 were analyzed by WB. (**D**) The *in vitro* kinase reactions containing purified f-Brd4, f-AFF1 or f-AFF4 together with dephosphorylated Cdk9-f and indicated peptides are shown as indicated. The levels of pT186 was analyzed by WB. (**E**) The phosphorylation status of Pol II and Spt5 was analyzed by WB in WCE from HeLa cells co-treated with HMBA and indicated peptides. (**F**) Luciferase activities were measured in extracts of cells stably transfected with the HIV-1 LTR-luciferase reporter gene after cotreatment with HMBA and peptides. The activity in untreated cells was set to 1.0. All values were expressed as Mean ± SD of three replicates. NS > 0.05, ***P < 0.001; *P*-values were assessed using two-tailed Student's *t*-test.

Since Cdk9 re-phosphorylation could be triggered without CycT1 (Figure 4A), it was very likely that T186 autophosphorylation resulted from the direct interactions between Cdk9 and its recruitment factors Brd4/AFF1/AFF4. To verify this, purified Cdk9 and CycT1 monomers were subjected to *in vitro* pull-down assay with immobilized HA-tagged Brd4, AFF1 or AFF4. Indeed, Cdk9 was pulled down by all three recruitment factors (Figures 5A, S5A and S5B). In contrast, CycT1 could only be pulled down when the core P-TEFb was reassembled (Figures 5A, S5A and S5B), indicating that Cdk9, but not CycT1, directly interacts with Brd4, AFF1 or AFF4.

Next, we determined the binding motif within Cdk9 for its interaction with Brd4 or AFF1/AFF4. As shown in Figure 3A, Cdk9-S175 mutants disrupted Cdk9′s interaction with Brd4, while Cdk9-T186 mutants failed to interact with AFF1/4, indicating that the T-loop of Cdk9 is the binding motif for Brd4/AFF1/AFF4. To test this hypothesis, a 20 a.a. long T-loop mimetic was designed (Supplementary Figure S5C) and expressed in HeLa cells to compete against Cdk9 for the binding of Brd4 or AFF1/AFF4. Consistently, we observed a significant decrease in Cdk9′s binding to Brd4 or SEC in the whole cell extract (WCE) of Cdk9 T-loop expressing cells after HMBA treatment (Figure 5B). We also observed a significant attenuation of Cdk9/CycT1 reassembly of core P-TEFb and T186 re-phosphorylation in these cells (Figure 5B). The ectopic expression of the T-loop peptide did not change the level of the factors tested (Supplementary Figure S5D). Furthermore, *in vitro* incubation with chemically synthesized T-loop peptides completely blocked Brd4- or AFF1/AFF4-induced re-phosphorylation of Cdk9 and the reassembly of core P-TEFb (Figures 5C and 5D). Consistent with this, expression of T-loop peptides in HeLa cells blocked HMBA-induced phosphorylation of Spt5 and Ser2 at the CTD of Pol II, two well-known targets of active P-TEFb (Figure 5E). Transcription of HIV-LTR-driven luciferase reporter gene, which is active P-TEFb-dependent, was also blocked by the expression of T-loop peptides (Figure 5F). These data suggest that the direct interaction between Brd4 or AFF1/AFF4 and Cdk9 T-loop is required for the reconstitution of active P-TEFb.

### The core P-TEFb undergoes a dissociation and reassembly process similar to the stress response during the cell cycle

**Figure 6.**
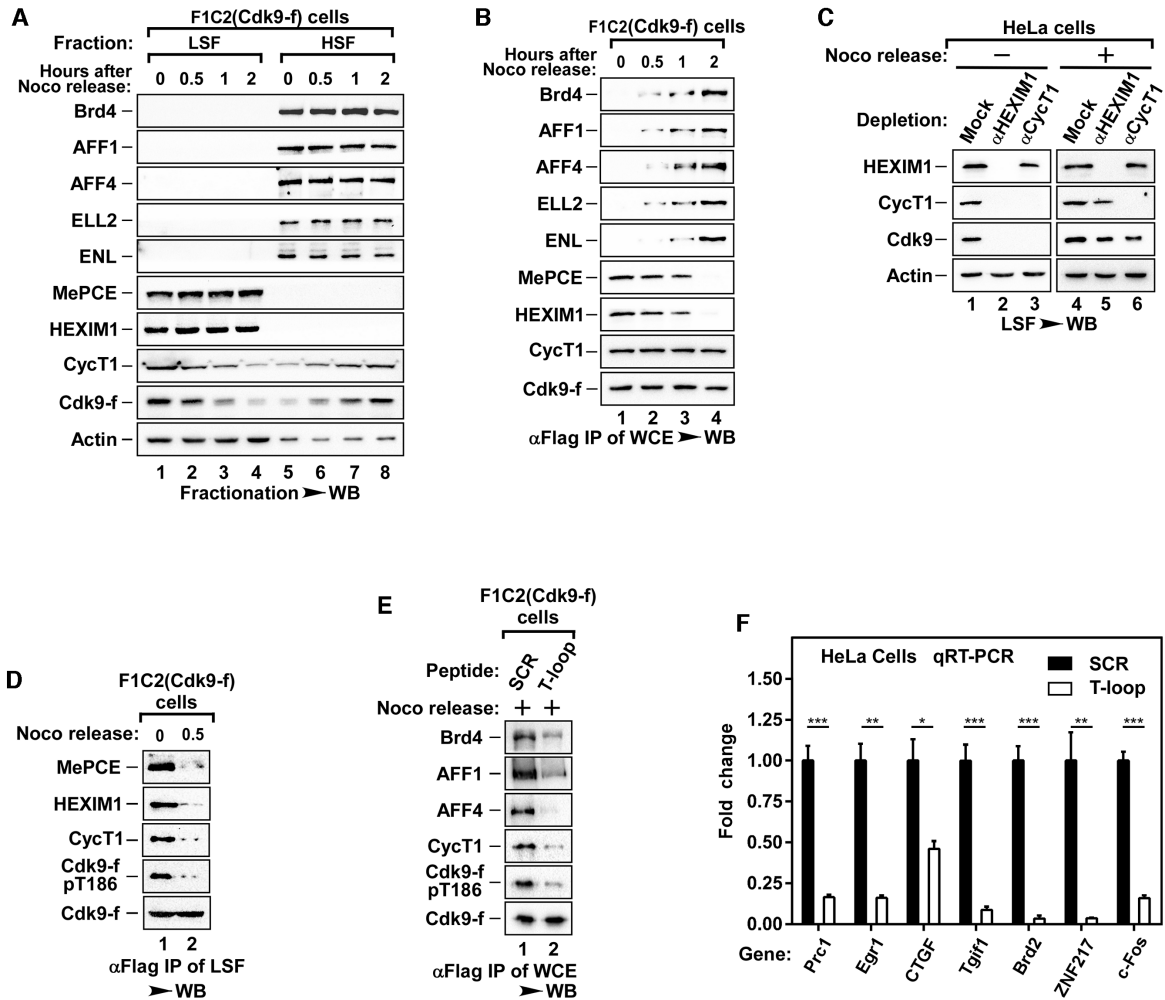
Brd4 and AFF1/AFF4 also facilitate the reconstitution of active P-TEFb from dissociated monomers during the cell cycle. (**A**) F1C2 cells were synchronized by nocodazole (Noco) treatment. Mitotic cells were collected by shake-off and replated in fresh medium (as Noco release). LSF and HSF were prepared at the indicated time points and then analyzed by WB for the indicated proteins. (**B**) Anti-Flag IPs of WCE from cells harvested at the indicated time points were analyzed by WB for the Cdk9-f-bound proteins. (**C**) LSF of synchronized HeLa cells was either mock- or immuno-depleted with immobilized anti-HEXIM1 or anti-CycT1 antibodies with or without Noco release. The levels of Cdk9, CycT1, HEXIM1, and Actin in the depleted LSF were analyzed by WB. (**D**) Synchronized F1C2 cells were released in fresh medium for 0.5 h. Anti-Flag IPs of LSF from cells was subjected to WB for the Cdk9-f-bound proteins. (**E**) Synchronized mitotic cells were released for 2 h in fresh medium containing the SCR or T-loop peptides. Anti-Flag IPs of WCE from cells were assayed as in B. (**F**) Total RNA isolated from Noco-released HeLa cells containing the SCR or T-loop peptides were analyzed by qRT-PCR for mRNA levels of representative G1 genes. The values for cells treated with SCR peptide was set to 1.00. All values are expressed as Mean ± SD of three replicates after normalization to 18S rRNA. *P < 0.05, **P < 0.01, ***P < 0.001; *P*-values were assessed using two-tailed Student's t-test.

Having demonstrated that P-TEFb undergoes heterodimer dissociation and T186 dephosphorylation, followed by reconstitution induced by Cdk9 binding to P-TEFb recruitment factors Brd4 or SEC in response to HMBA or stress, we next explored whether other cellular processes would also employ similar mechanism. Transcription is tightly regulated during cell cycle as transcription is mostly shut down in M phase, and the compacting chromatins displace active P-TEFb (21). Upon entry into early G1, transcription is restarted, likely accompanied by P-TEFb activation from a completely inactive state. To assess whether cells use similar mechanism for the re-activation of P-TEFb during the cell cycle, we synchronized cells in mitosis by treating HeLa cells with nocodazole, a drug blocking cells from entering early G1 phase. Examining the P-TEFb distribution in fractionated LSF and HSF of these mitotic cells, we found that nearly all P-TEFb was in the LSF (Figure 6A lanes 1 and 5), a significant distribution difference compared with freely dividing cells (Figure 1B, lanes 1 and 3). Release of nocodazole drove the cell cycle into early G1 phase with the *de novo* P-TEFb activation (Figure 6B). More P-TEFb was recruited to the HSF in a time-dependent manner (Figure 6A, lanes 5–8). The migration of P-TEFb peaked at 2 h after nocodazole release, with nearly all P-TEFb now distributed in the HSF, suggesting that the majority of P-TEFb was recruited to the chromatin (Figure 6A). Furthermore, immunoprecipitation of Cdk9-f from WCE of F1C2 cells revealed that P-TEFb transitioned from the 7SK snRNP complex into the Brd4 and SEC complexes with the release of nocodazole (Figure 6B). Notably, there were no changes in the expression of all the indicated proteins during nocodazole release (Supplementary Figure S6A).

The activation of P-TEFb during early G1 entry also required dissociation of Cdk9 and CycT1 heterodimer as well as the dephosphorylation of T186. When the LSF of either nocodazole-induced mitotic cells or nocodazole-released early G1 cells were subjected to HEXIM1 depletion, we observed that HEXIM1 completely co-depleted P-TEFb in mitotic cells (Figure 6C), further confirming that the majority of P-TEFb was indeed sequestered in 7SK snRNP. However, depletion of CycT1 failed to co-deplete Cdk9 from nocodazole released cells (Figure 6C, lane 6), suggesting the dissociation of core P-TEFb in these nocodazole released cells. Immunoprecipitation of Cdk9 also demonstrated the dissociation of CycT1 from Cdk9, as well as the dephosphorylation of T186 during nocodazole release, similar to HMBA or stress treatment (Figure 6D).

We next examined whether the reconstitution of P-TEFb during nocodazole release also utilized the similar mechanism as HMBA or stress-induced reconstitution. When T-loop peptides were introduced to nocodazole releasing cells, Cdk9 monomers failed to bind to Brd4 or SEC, subsequently blocking the reconstitution of active P-TEFb (Figure 6E and Supplementary Figure S6B). With the inhibition of reconstitution by the T-loop peptides, the recruitment of Cdk9 towards the promoters of typical early G1 genes (23,28) was reduced (Supplementary Figure S6C), resulting in a significant decrease in the expression of these genes (Figure 6F and Supplementary Figure S6C). All together, these data demonstrate that during transition into early G1, the *de novo* activation of P-TEFb also requires the dissociation of core P-TEFb, dephosphorylation of pT186, and the reconstitution of active P-TEFb.

## DISCUSSION

**Figure 7.**
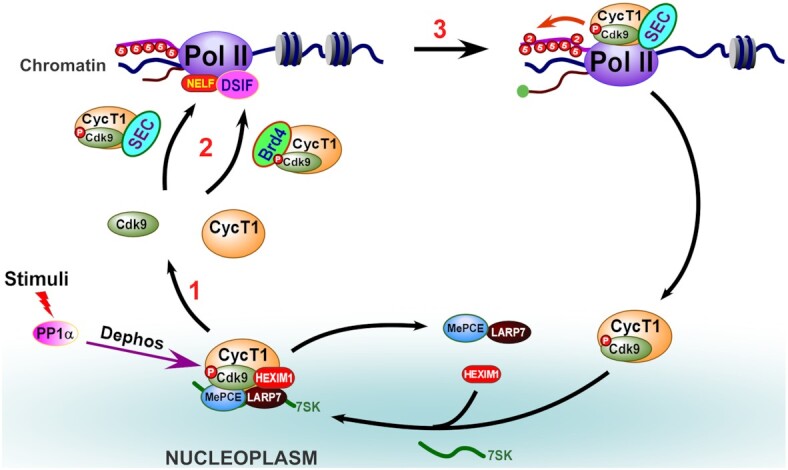
Schematic model for the dissociation and reconstitution of P-TEFb. 1) Stress or entry into early G1 stimulates PP1α-mediated dephosphorylation of Cdk9-pT186, leading to disruption of 7SK snRNP and dissociation of the core P-TEFb. 2) The dissociated Cdk9 and CycT1 monomers are then reconstituted via direct interaction with Brd4 or SEC through the Cdk9 T-loop, accompanied by the re-phosphorylation of T186. 3) The reconstituted active P-TEFb then activates inducible genes via the release of Pol II.

In cells, P-TEFb is mostly found reserved in the inactive 7SK snRNP complex, P-TEFb is activated by its release and recruitment by Brd4 and SEC in response to stress and other cellular activities. Based on our previous reports (24), the release of core P-TEFb from 7SK snRNP occurs via activation by protein phosphatase PP2B and PP1α signaling pathways. However, how the P-TEFb is released and reassembled into its active form remains unclear. Here, we demonstrate that the release of core P-TEFb is accompanied by its dissociation into Cdk9 and CycT1 monomers facilitated by PP1α dephosphorylation of pT186. Following this, the monomerized Cdk9 is reassembled with CycT1 into core P-TEFb, mediated by its interaction with Brd4 or AFF1/AFF4 through the T-loop. Meanwhile, this interaction directly stimulates the autophosphorylation of Cdk9 at T186, which eventually leads the reconstitution of active P-TEFb and the activation of inducible genes (Figure 7).

The observation that the core P-TEFb of 7SK snRNP was further dissociated under various stresses and mitotic conditions was intriguing. While the dissociation of core P-TEFb has previously been reported under various conditions (29–31), which contributed to the decrease of P-TEFb activity, we observed here that the dissociation of core P-TEFb was a necessary step during stress/cell cycle induced P-TEFb activation, as disruption of following reconstitution step resulted in nearly all Cdk9 in the cells being monomers (Figures 5B and 6E). During the past decade, several pathways leading to the disassembly of 7SK snRNP have been elucidated (24,32–34), which are believed to activate transcription by releasing core P-TEFb. Since HEXIM1 along with scaffold 7SK inhibits the Cdk9 kinase, it is reasonable that the interactions of these three components are targeted to perturb the integrity of 7SK snRNP. For example, stress-induced phosphorylation of HEXIM1 by protein kinase C (PKC) affects its interaction with 7SK (32). Alternatively, DRB or ActD treatment atypically stimulates the release of HEXIM1 through competitive binding of 7SK with the hnRNPs (33,34). Another account is the promotion of the conformational changes of 7SK snRNP by PP2B through a yet-to-be-identified target, which exposes pT186 for PP1α. When this occurs, PP1α dephosphorylates pT186 and detaches the phosphorylation-dependent binding of scaffold 7SK to Cdk9 (24). Notably, once core P-TEFb deviates from HEXIM1, there would be inevitable dephosphorylation of pT186, and therefore the simultaneous destruction of the Cdk9/CycT1 heterodimer (Figure 2D). These observations could be the reason for the dissociation of core P-TEFb under various stresses (Supplementary Figure S1B), in cells from different tissues (Supplementary Figure S1C), and even during mitosis (Figure 6).

Our study also elucidates the underlying mechanism by which P-TEFb is reconstituted into full transcriptional activity after release from 7SK snRNP. We have observed here that T186 underwent autophosphorylation in an interaction-dependent manner (Figure 4). In fact, our results indicate that almost all active P-TEFb was reconstituted through interaction with either recruitment factors Brd4 or AFF1/AFF4 (Figure 3E). As long as Cdk9 binds to Brd4 or AFF1/AFF4, T186 autophosphorylation occurs *in vitro* (Figures 5A and 5B). Moreover, these key interactions were contingent on the binding of Cdk9 T-loop to these recruitment factors rather than CycT1 (35) or other SEC components (Figure 5). Similarly, pretreatment with T-loop peptides abrogated the reconstitution of active P-TEFb, thereby attenuating the phosphorylation of Pol II-Ser2 and the transcription of P-TEFb-dependent HIV-LTR-luciferase (Figure 5).

Another interesting aspect of the reconstitution mechanism is that the restoration of the Cdk9/CycT1 heterodimer and the autophosphorylation of T186 are two independent events, which further ensure that the core P-TEFb recruited by Brd4 or SEC will not be improperly destroyed by PP1α before its recruitment to gene promoters. Based on these, we are convinced that core P-TEFb is recovered before being recruited toward chromatin, and that T186 autophosphorylation is not required at the same time, an observation consistent with our previous reports (24). Another possible benefit of reonstitution of active P-TEFb with two independent events comes from the diverse ways to phosphorylate T186. T186 autophosphorylation was firstly revealed when studying the structure of the core P-TEFb (36). Later, the CaMK1D (Ca^2+^/Calmodulin dependent Kinase 1D) was identified as a candidate for T186 kinase from studies of calcium signaling in HeLa cells and primary CD^4+^ T lymphocytes (37). Almost at the same time, Cdk7 was identified as a T186 kinase in HCT116 cells induced by specific chemical (38). Therefore, similar to the various pathways that disrupts 7SK snRNP to release P-TEFb, different T186 phosphorylation pathways can be utilized to accommodate corresponding stimuli. In combination with our previous reports, during HMBA-induced stress or cell cycle release, T186 autophosphorylation is the dominant mechanism to efficiently reconstitute P-TEFb and release the promoter proximally paused Pol II (18) or restart global closed transcription, respectively.

The constitution of active P-TEFb from inactive 7SK snRNP had always been studied under stress conditions, with studies rarely exploring the activation of P-TEFb under physiological processes such as the cell cycle. Given that the expression level of P-TEFb remains constant during the cell cycle, its transcriptional control of early G1 genes through Brd4-dependent recruitment serves as the only mechanism to regulate cell cycle progression (21–23). Our current data uncover another mechanism, where the P-TEFb is almost completely sequestered in 7SK snRNP during mitosis, requiring *de novo* activation through dissociation of the Cdk9/CycT1 heterodimer (Figure 6). Importantly, these observations are consistent with the overall transcriptional shutdown in cells during mitosis (21). Treatment of cells in mitosis with Cdk9 T-loop peptides significantly impaired the transcription of early G1 genes (Figure 6). Thus, our current study provides new insights into the role of P-TEFb and its associated factors in the control of the cell cycle.

In conclusion, our data demonstrated a delicate process whereby signals induce the complete dissociation of inactive 7SK snRNP to release monomeric Cdk9 and CycT1, which then are reconstituted back to active P-TEFb facilitated by Brd4 and SEC to enhance the transcription of inducible genes. It would be of great interest to determine whether the mechanism identified here would also apply to the transcriptional regulation of other P-TEFb-dependent genes in other biological settings.

## Supplementary Material

gkab1228_Supplemental_File

## References

[1] Chen F.X. , Smith E.R., Shilatifard A. Born to run: control of transcription elongation by RNA polymerase II. Nat. Rev. Mol. Cell Biol. 2018; 19:464–478. 29740129 10.1038/s41580-018-0010-5

[2] Li Y. , Liu M., Chen L.F., Chen R. P-TEFb: finding its ways to release promoter-proximally paused RNA polymerase II. Transcription. 2018; 9:88–94. 28102758 10.1080/21541264.2017.1281864 PMC5834220

[3] Jonkers I. , Lis J.T. Getting up to speed with transcription elongation by RNA polymerase II. Nat. Rev. Mol. Cell Biol. 2015; 16:167–177. 25693130 10.1038/nrm3953 PMC4782187

[4] Zeitlinger J. , Stark A., Kellis M., Hong J.W., Nechaev S., Adelman K., Levine M., Young R.A. RNA polymerase stalling at developmental control genes in the Drosophila melanogaster embryo. Nat. Genet. 2007; 39:1512–1516. 17994019 10.1038/ng.2007.26 PMC2824921

[5] Muse G.W. , Gilchrist D.A., Nechaev S., Shah R., Parker J.S., Grissom S.F., Zeitlinger J., Adelman K. RNA polymerase is poised for activation across the genome. Nat. Genet. 2007; 39:1507–1511. 17994021 10.1038/ng.2007.21 PMC2365887

[6] Yamaguchi Y. , Shibata H., Handa H. Transcription elongation factors DSIF and NELF: promoter-proximal pausing and beyond. Biochim. Biophys. Acta. 2013; 1829:98–104. 23202475 10.1016/j.bbagrm.2012.11.007

[7] Nechaev S. , Fargo D.C., dos Santos G., Liu L., Gao Y., Adelman K. Global analysis of short RNAs reveals widespread promoter-proximal stalling and arrest of Pol II in Drosophila. Science. 2010; 327:335–338. 20007866 10.1126/science.1181421 PMC3435875

[8] Core L.J. , Waterfall J.J., Lis J.T. Nascent RNA sequencing reveals widespread pausing and divergent initiation at human promoters. Science. 2008; 322:1845–1848. 19056941 10.1126/science.1162228 PMC2833333

[9] Chen R. , Yik J.H., Lew Q.J., Chao S.H. Brd4 and HEXIM1: multiple roles in P-TEFb regulation and cancer. Biomed. Res. Int. 2014; 2014:232870. 24592384 10.1155/2014/232870 PMC3925632

[10] Bacon C.W. , D’Orso I CDK9: a signaling hub for transcriptional control. Transcription. 2019; 10:57–75. 30227759 10.1080/21541264.2018.1523668 PMC6602564

[11] Yik J.H. , Chen R., Nishimura R., Jennings J.L., Link A.J., Zhou Q. Inhibition of P-TEFb (CDK9/Cyclin T) kinase and RNA polymerase II transcription by the coordinated actions of HEXIM1 and 7SK snRNA. Mol. Cell. 2003; 12:971–982. 14580347 10.1016/s1097-2765(03)00388-5

[12] Chen R. , Yang Z., Zhou Q. Phosphorylated positive transcription elongation factor b (P-TEFb) is tagged for inhibition through association with 7SK snRNA. J. Biol. Chem. 2004; 279:4153–4160. 14627702 10.1074/jbc.M310044200

[13] Yik J.H. , Chen R., Pezda A.C., Zhou Q. Compensatory contributions of HEXIM1 and HEXIM2 in maintaining the balance of active and inactive positive transcription elongation factor b complexes for control of transcription. J. Biol. Chem. 2005; 280:16368–16376. 15713661 10.1074/jbc.M500912200

[14] He N. , Jahchan N.S., Hong E., Li Q., Bayfield M.A., Maraia R.J., Luo K., Zhou Q. A La-related protein modulates 7SK snRNP integrity to suppress P-TEFb-dependent transcriptional elongation and tumorigenesis. Mol. Cell. 2008; 29:588–599. 18249148 10.1016/j.molcel.2008.01.003 PMC6239424

[15] Xue Y. , Yang Z., Chen R., Zhou Q. A capping-independent function of MePCE in stabilizing 7SK snRNA and facilitating the assembly of 7SK snRNP. Nucleic Acids Res. 2010; 38:360–369. 19906723 10.1093/nar/gkp977 PMC2811026

[16] Yang Z. , Yik J.H., Chen R., He N., Jang M.K., Ozato K., Zhou Q. Recruitment of P-TEFb for stimulation of transcriptional elongation by the bromodomain protein Brd4. Mol. Cell. 2005; 19:535–545. 16109377 10.1016/j.molcel.2005.06.029

[17] He N. , Liu M., Hsu J., Xue Y., Chou S., Burlingame A., Krogan N.J., Alber T., Zhou Q. HIV-1 Tat and host AFF4 recruit two transcription elongation factors into a bifunctional complex for coordinated activation of HIV-1 transcription. Mol. Cell. 2010; 38:428–438. 20471948 10.1016/j.molcel.2010.04.013 PMC3085314

[18] Lu X. , Zhu X., Li Y., Liu M., Yu B., Wang Y., Rao M., Yang H., Zhou K., Wang Y. et al. Multiple P-TEFbs cooperatively regulate the release of promoter-proximally paused RNA polymerase II. Nucleic Acids Res. 2016; 44:6853–6867. 27353326 10.1093/nar/gkw571 PMC5001612

[19] He N. , Chan C.K., Sobhian B., Chou S., Xue Y., Liu M., Alber T., Benkirane M., Zhou Q. Human Polymerase-Associated Factor complex (PAFc) connects the Super Elongation Complex (SEC) to RNA polymerase II on chromatin. Proc. Natl. Acad. Sci. U.S.A. 2011; 108:E636–E645. 21873227 10.1073/pnas.1107107108 PMC3169135

[20] Garriga J. , Bhattacharya S., Calbo J., Marshall R.M., Truongcao M., Haines D.S., Grana X. CDK9 is constitutively expressed throughout the cell cycle, and its steady-state expression is independent of SKP2. Mol. Cell Biol. 2003; 23:5165–5173. 12861003 10.1128/MCB.23.15.5165-5173.2003 PMC165719

[21] Yang Z. , He N., Zhou Q. Brd4 recruits P-TEFb to chromosomes at late mitosis to promote G1 gene expression and cell cycle progression. Mol. Cell Biol. 2008; 28:967–976. 18039861 10.1128/MCB.01020-07 PMC2223388

[22] Mochizuki K. , Nishiyama A., Jang M.K., Dey A., Ghosh A., Tamura T., Natsume H., Yao H., Ozato K. The bromodomain protein Brd4 stimulates G1 gene transcription and promotes progression to S phase. J. Biol. Chem. 2008; 283:9040–9048. 18223296 10.1074/jbc.M707603200 PMC2431025

[23] Dey A. , Nishiyama A., Karpova T., McNally J., Ozato K. Brd4 marks select genes on mitotic chromatin and directs postmitotic transcription. Mol. Biol. Cell. 2009; 20:4899–4909. 19812244 10.1091/mbc.E09-05-0380 PMC2785733

[24] Chen R. , Liu M., Li H., Xue Y., Ramey W.N., He N., Ai N., Luo H., Zhu Y., Zhou N. et al. PP2B and PP1alpha cooperatively disrupt 7SK snRNP to release P-TEFb for transcription in response to Ca2+ signaling. Genes Dev. 2008; 22:1356–1368. 18483222 10.1101/gad.1636008 PMC2377190

[25] Hu X. , Lu X., Liu R., Ai N., Cao Z., Li Y., Liu J., Yu B., Liu K., Wang H. et al. Histone cross-talk connects protein phosphatase 1alpha (PP1alpha) and histone deacetylase (HDAC) pathways to regulate the functional transition of bromodomain-containing 4 (BRD4) for inducible gene expression. J. Biol. Chem. 2014; 289:23154–23167. 24939842 10.1074/jbc.M114.570812 PMC4132813

[26] Ai N. , Hu X., Ding F., Yu B., Wang H., Lu X., Zhang K., Li Y., Han A., Lin W. et al. Signal-induced Brd4 release from chromatin is essential for its role transition from chromatin targeting to transcriptional regulation. Nucleic Acids Res. 2011; 39:9592–9604. 21890894 10.1093/nar/gkr698 PMC3239188

[27] Devaiah B.N. , Lewis B.A., Cherman N., Hewitt M.C., Albrecht B.K., Robey P.G., Ozato K., Sims R.J. 3rd, Singer D.S. BRD4 is an atypical kinase that phosphorylates serine2 of the RNA polymerase II carboxy-terminal domain. Proc. Natl. Acad. Sci. U.S.A. 2012; 109:6927–6932. 22509028 10.1073/pnas.1120422109 PMC3345009

[28] Beyrouthy M.J. , Alexander K.E., Baldwin A., Whitfield M.L., Bass H.W., McGee D., Hurt M.M. Identification of G1-regulated genes in normally cycling human cells. PLoS One. 2008; 3:e3943. 19079774 10.1371/journal.pone.0003943 PMC2600614

[29] Charles S. , Ammosova T., Cardenas J., Foster A., Rotimi J., Jerebtsova M., Ayodeji A.A., Niu X., Ray P.E., Gordeuk V.R. et al. Regulation of HIV-1 transcription at 3% versus 21% oxygen concentration. J. Cell Physiol. 2009; 221:469–479. 19626680 10.1002/jcp.21882 PMC2778305

[30] Debebe Z. , Ammosova T., Breuer D., Lovejoy D.B., Kalinowski D.S., Kumar K., Jerebtsova M., Ray P., Kashanchi F., Gordeuk V.R. et al. Iron chelators of the di-2-pyridylketone thiosemicarbazone and 2-benzoylpyridine thiosemicarbazone series inhibit HIV-1 transcription: identification of novel cellular targets–iron, cyclin-dependent kinase (CDK) 2, and CDK9. Mol. Pharmacol. 2011; 79:185–196. 20956357 10.1124/mol.110.069062 PMC3014282

[31] Yu D. , Cattoglio C., Xue Y., Zhou Q. A complex between DYRK1A and DCAF7 phosphorylates the C-terminal domain of RNA polymerase II to promote myogenesis. Nucleic Acids Res. 2019; 47:4462–4475. 30864669 10.1093/nar/gkz162 PMC6511856

[32] Fujinaga K. , Barboric M., Li Q., Luo Z., Price D.H., Peterlin B.M. PKC phosphorylates HEXIM1 and regulates P-TEFb activity. Nucleic Acids Res. 2012; 40:9160–9170. 22821562 10.1093/nar/gks682 PMC3467075

[33] Van Herreweghe E. , Egloff S., Goiffon I., Jady B.E., Froment C., Monsarrat B., Kiss T. Dynamic remodelling of human 7SK snRNP controls the nuclear level of active P-TEFb. EMBO J. 2007; 26:3570–3580. 17611602 10.1038/sj.emboj.7601783 PMC1949012

[34] Barrandon C. , Bonnet F., Nguyen V.T., Labas V., Bensaude O. The transcription-dependent dissociation of P-TEFb-HEXIM1-7SK RNA relies upon formation of hnRNP-7SK RNA complexes. Mol. Cell Biol. 2007; 27:6996–7006. 17709395 10.1128/MCB.00975-07 PMC2168891

[35] Schroder S. , Cho S., Zeng L., Zhang Q., Kaehlcke K., Mak L., Lau J., Bisgrove D., Schnolzer M., Verdin E. et al. Two-pronged binding with bromodomain-containing protein 4 liberates positive transcription elongation factor b from inactive ribonucleoprotein complexes. J. Biol. Chem. 2012; 287:1090–1099. 22084242 10.1074/jbc.M111.282855 PMC3256921

[36] Baumli S. , Lolli G., Lowe E.D., Troiani S., Rusconi L., Bullock A.N., Debreczeni J.E., Knapp S., Johnson L.N. The structure of P-TEFb (CDK9/cyclin T1), its complex with flavopiridol and regulation by phosphorylation. EMBO J. 2008; 27:1907–1918. 18566585 10.1038/emboj.2008.121 PMC2486423

[37] Ramakrishnan R. , Rice A.P. Cdk9 T-loop phosphorylation is regulated by the calcium signaling pathway. J. Cell Physiol. 2012; 227:609–617. 21448926 10.1002/jcp.22760 PMC3146566

[38] Larochelle S. , Amat R., Glover-Cutter K., Sanso M., Zhang C., Allen J.J., Shokat K.M., Bentley D.L., Fisher R.P. Cyclin-dependent kinase control of the initiation-to-elongation switch of RNA polymerase II. Nat. Struct. Mol. Biol. 2012; 19:1108–1115. 23064645 10.1038/nsmb.2399 PMC3746743

