# Supplementary material for: Disrupting the Cdk9/Cyclin T1 heterodimer of 7SK snRNP for the Brd4 and AFF1/4 guided reconstitution of active P-TEFb
Source: Nucleic Acids Res. 2021 Dec 22;50(2):750–62. doi: 10.1093/nar/gkab1228 (PMC8789079; doi:10.1093/nar/gkab1228)
Supplement: gkab1228_Supplemental_File [file gkab1228_supplemental_file.pdf]

Supplementary information

Disrupting the Cdk9/Cyclin T1 Heterodimer of 7SK snRNP for the Brd4 and AFF1/4 Guided Reconstitution of Active P-TEFb

Kai Zhou[1#], Songkuan Zhuang[1#], Fulong Liu[1#], Yanheng Chen[2#], You Li[3], Shihui Wang[1], Yuxuan Li[1], Huixin Wen[1], Xiaohua Lin[1], Jie Wang[1], Yue Huang[1], Cailing He[1], Nan Xu[1], Zongshu Li[1], Lang Xu[1], Zixuan Zhang[1], Lin-Feng Chen[2*], Ruichuan Chen[1*], Min Liu[1*].

[1] State Key Laboratory of Cellular Stress Biology, School of Life Sciences, Xiamen University, Xiamen 361005, Fujian, China.

[2] Department of Biochemistry, University of Illinois at Urbana-Champaign, Urbana, IL 61801, USA.

[3] Biomolecular Interaction Centre, University of Canterbury, Christchurch 8140, New Zealand

[#]These authors contributed equally to this work

[*]Correspondence: minliu@xmu.edu.cn; chenrc@xmu.edu.cn; lfchen@illinois.edu

Including:

Supplementary Figure S1-S5 and Supplementary Materials and Methods.

Fig. S1

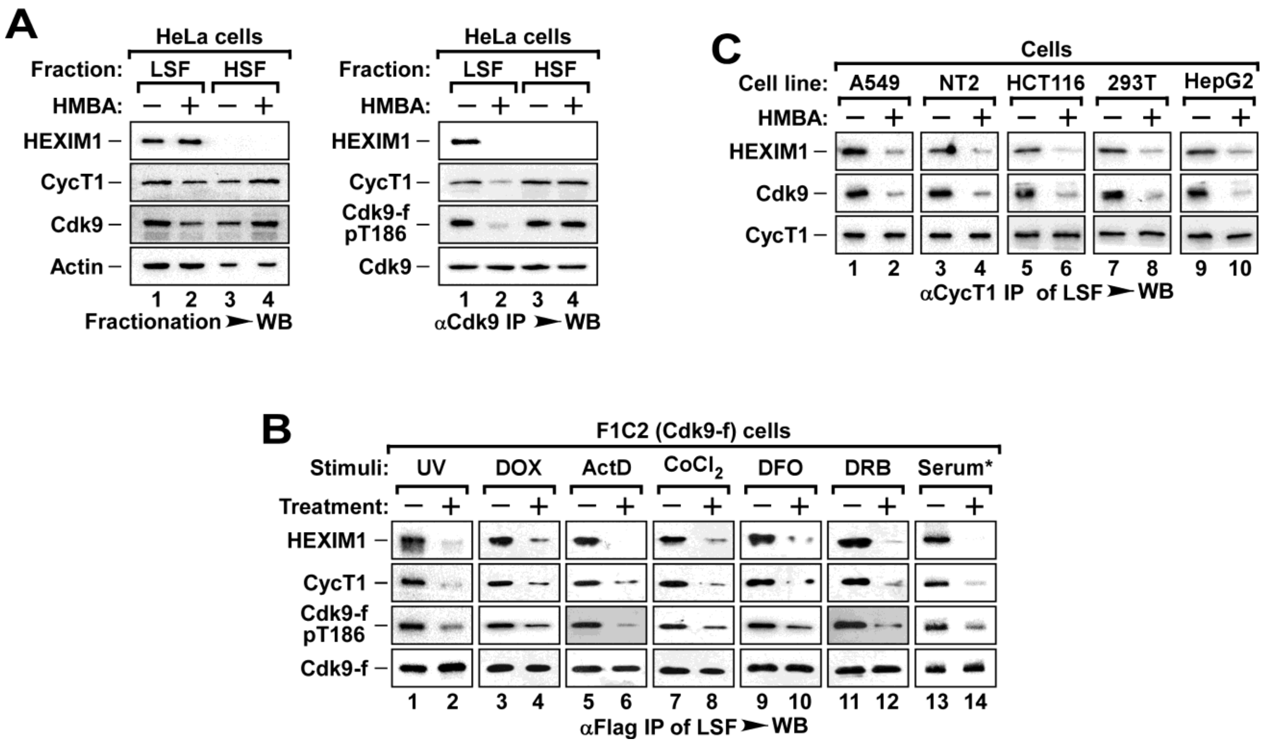

Figure S1. The stress-induced dissociation of core P-TEFb is a common feature in multiple cell lines. (**A**) The LSF and HSF were prepared from HeLa cells treated with or without HMBA. The levels of indicated proteins in the fractions (left panel) or anti-CDK9 IPs (right panel) were analyzed by WB. (**B**) F1C2 cells were treated with ultraviolet (UV, 80J/m^2), doxorubicin (DOX, 5 μg/ml), actinomycin D (ActD, 1 μg/ml), $CoCl_2$ (100 μM) or Desferrioxamine (DFO, 100 μM) for 1 h, or treated with 5,6-dichloro-1-b-D-ribofuranosyl-benzimidazole (DRB, 50 μM) or serum-free medium (Serum*) for 2 h. Anti-Flag IPs from LSF were subjected to WB for the levels of pT186 and Cdk9-f-bound proteins. (**C**) Anti-CycT1 IPs derived from LSF of different cells, including A549, NT2, HCT116, 293T or HepG2 cells, were analyzed by WB with indicated antibodies.

Fig. S2

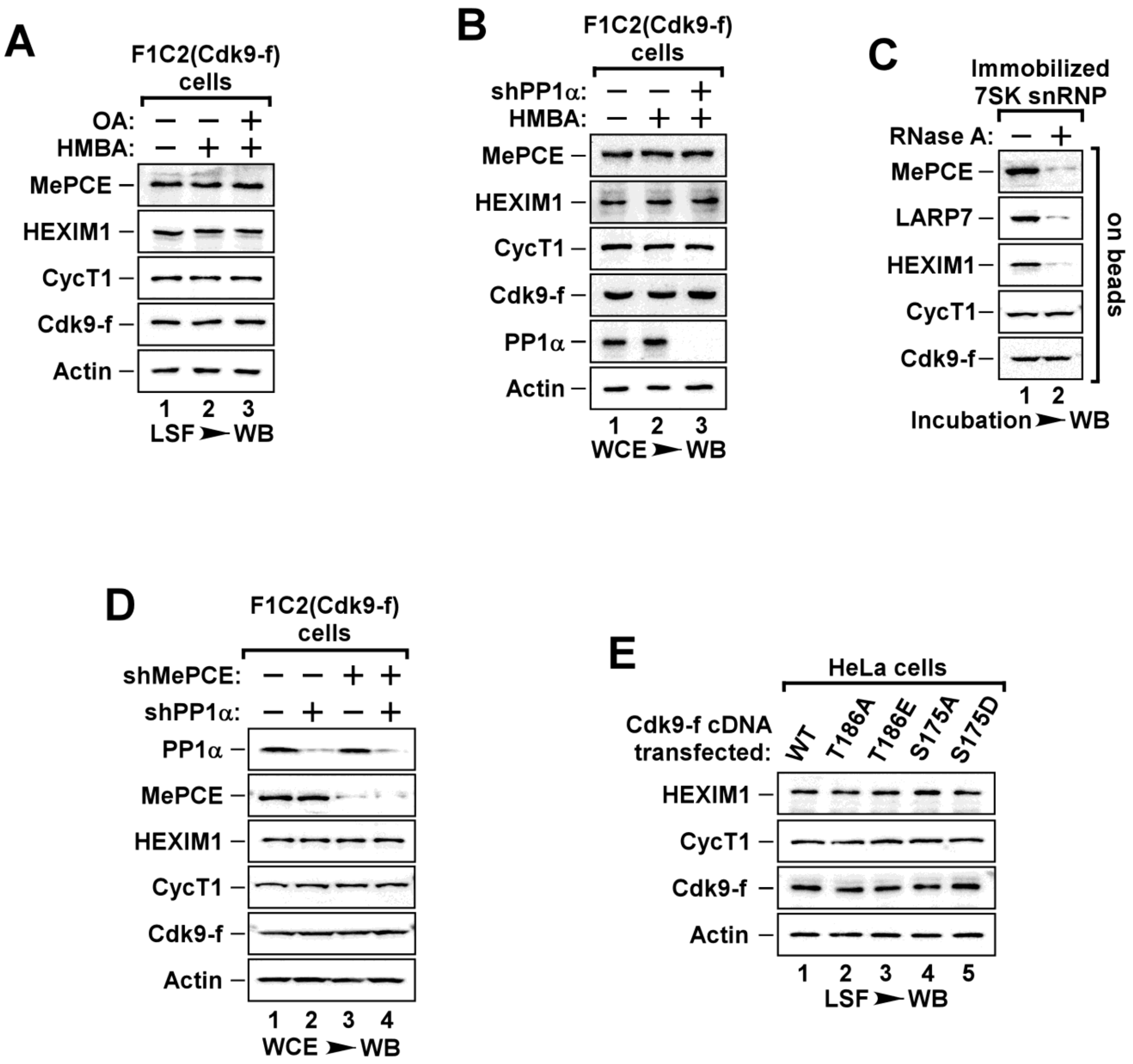

Figure S2. PP1α-mediated dephosphorylation of pT186 directly induces the dissociation of core P-TEFb. (**A**) The expression levels of indicated proteins from F1C2 LSF treated with Okadaic acid (OA) and HMBA were analyzed by WB. (**B**) The levels of indicated proteins from F1C2 WCE treated with shPP1α and HMBA were analyzed by WB. (**C**) Affinity-purified P-TEFb from LSF of F1C2 cells was incubated with RNase A. The levels of Cdk9-f-associated proteins were analyzed by WB. (**D**) The levels of indicated proteins from F1C2 WCE infected with shPP1α and shMePCE were analyzed by WB. (**E**) The expression levels of indicated proteins from HeLa LSF transfected with various mutants of Cdk9-f were analyzed by WB.

Fig. S3

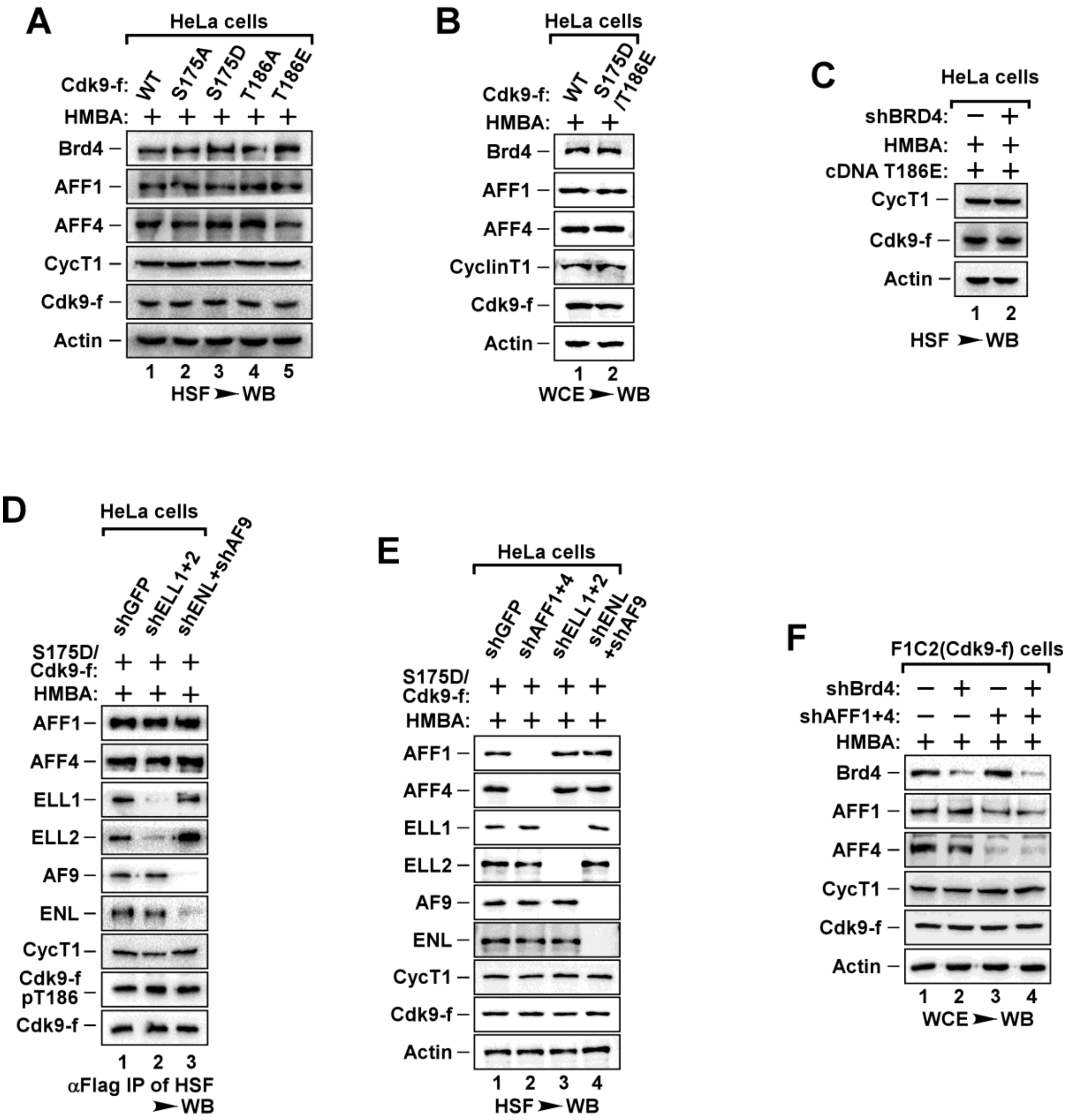

Figure S3. Brd4 and AFF1/4 facilitate the reassembly of core P-TEFb from dissociated monomers. (**A & B**) The levels of indicated proteins from HeLa HSF or WCE transfected with various mutants of Cdk9-f were analyzed by WB. All cells in (A) to (F) were treated with HMBA before harvesting. (**C**) The levels of indicated proteins from HeLa HSF transfected with T186E-Cdk9-f and shBrd4 were analyzed by WB. (**D**) Anti-Flag IPs from HeLa HSF transfected with S175D-Cdk9-f and indicated shRNAs were analyzed by WB. (**E**) WB analysis of the expression levels of indicated proteins from HeLa HSF infected with S175D-Cdk9-f and indicated shRNAs. (**F**) WB analysis of the expression levels of indicated proteins from F1C2 WCE transfected with shBrd4, shAFF1 and shAFF4.

Fig. S4

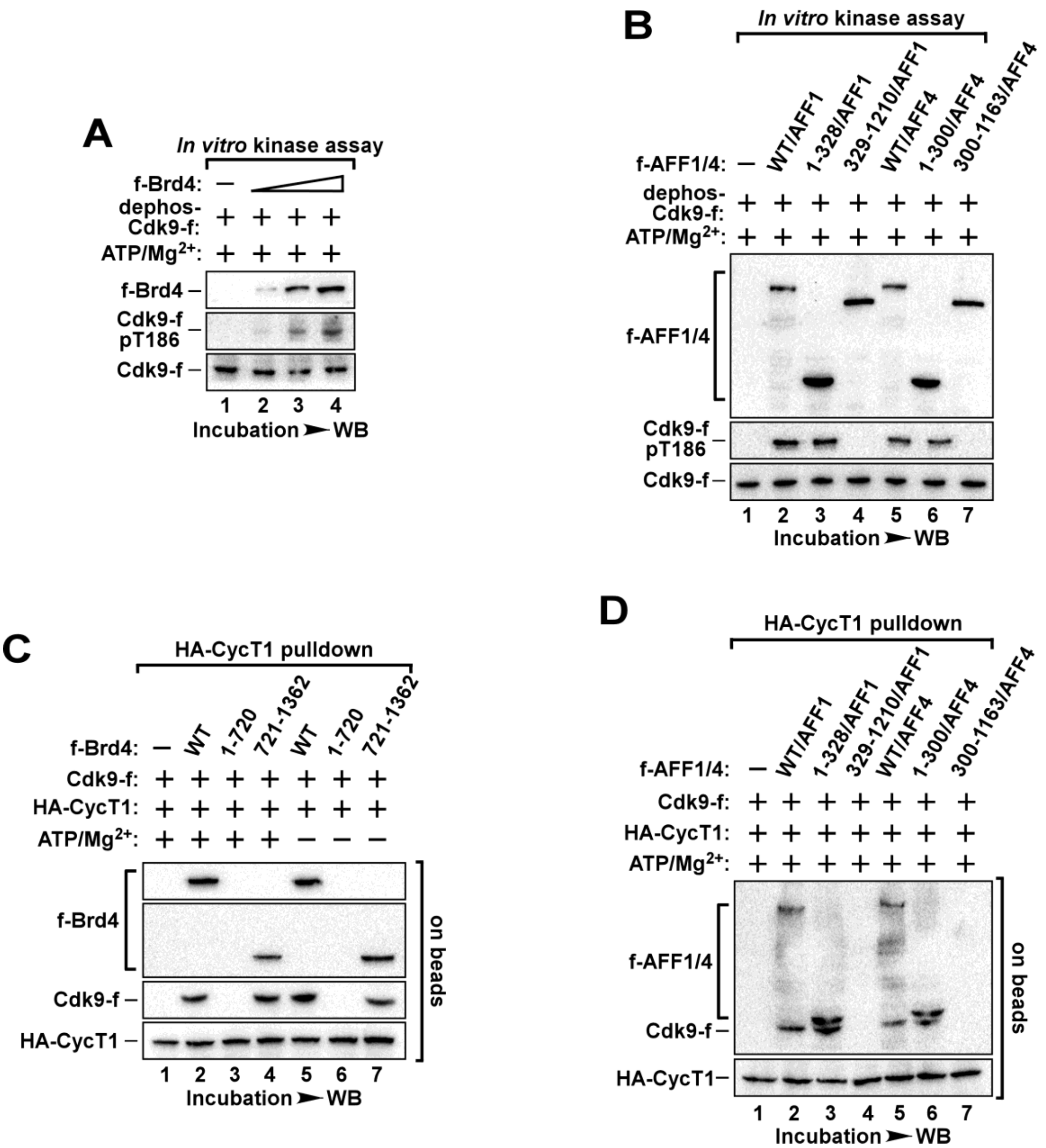

Figure S4. Brd4 and AFF1/4 mediate the autophosphorylation of Cdk9-T186. (A) Brd4 directly facilitated the autophosphorylation of monomeric Cdk9 at T186 in a dose-dependent manner. WB analysis of pT186 levels in kinase reactions with dephosphorylated Cdk9-f (dephos-Cdk9-f) and increased f-Brd4. (**B**) WB analysis of pT186 levels in kinase reactions containing dephos-Cdk9-f and full-length or truncated f-AFF1/4 as indicated. (**C**) WB analysis of the levels of Cdk9-f bound to immobilized HA-CycT1 in the presence of full-length or truncated Brd4 with/without ATP/Mg^{2+}. (**D**) WB analysis of the levels of Cdk9-f bound to immobilized HA-CycT1 in pull-down reactions containing indicated proteins with ATP/Mg^{2+}.

Fig. S5

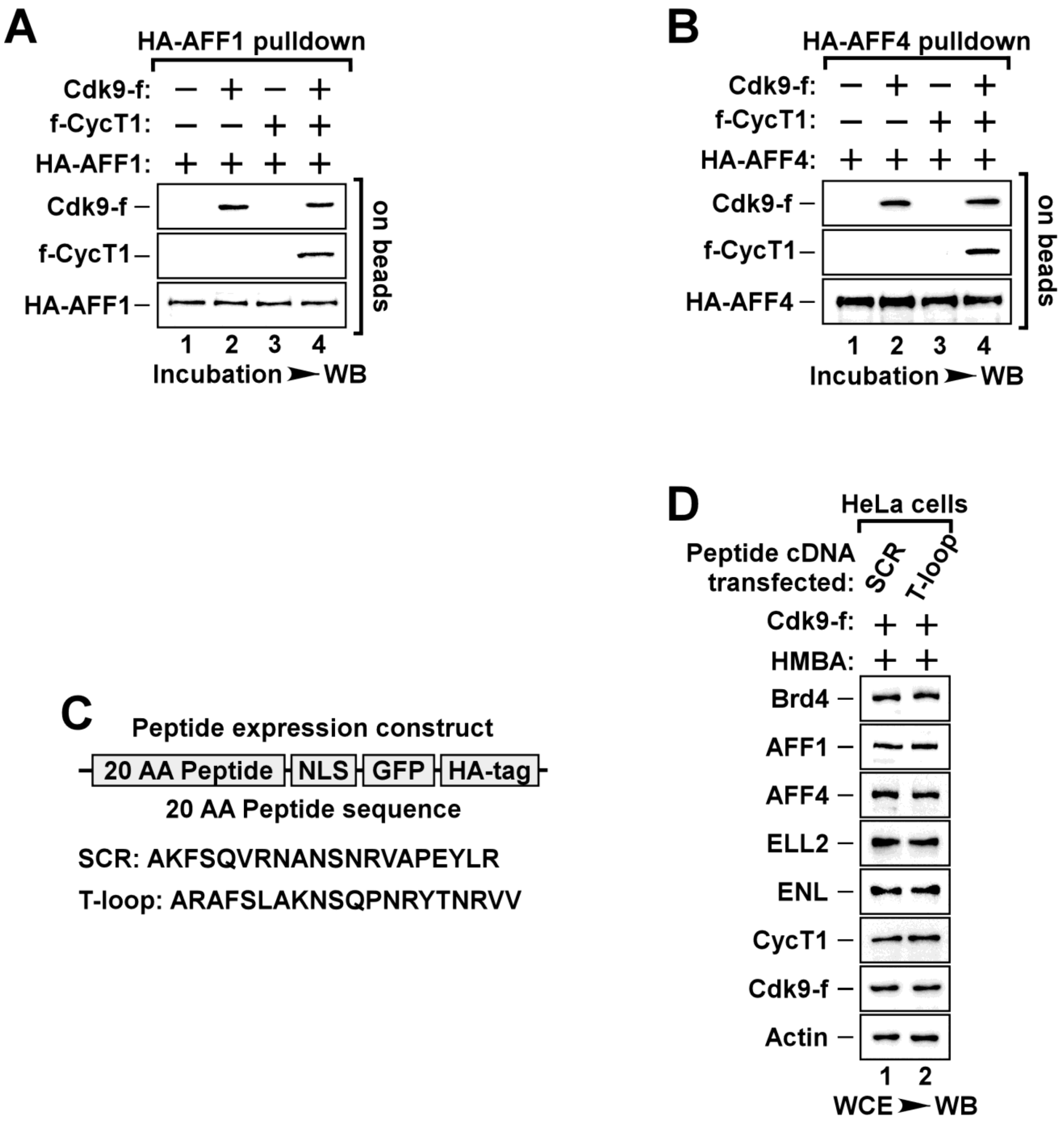

Figure S5. Cdk9 T-loop peptide blocks Brd4/AFF1/AFF4-mediated reconstitution of active P-TEFb. (**A & B**) WB analysis of the levels of Cdk9-f and f-CycT1 bound to immobilized HA-AFF1 or HA-AFF4. (**C**) Sketch map of peptide expression constructs. (**D**) WB analysis of the expression levels of indicated proteins in HMBA treated HeLa WCE cotransfected with Cdk9-f and peptide.

Fig. S6

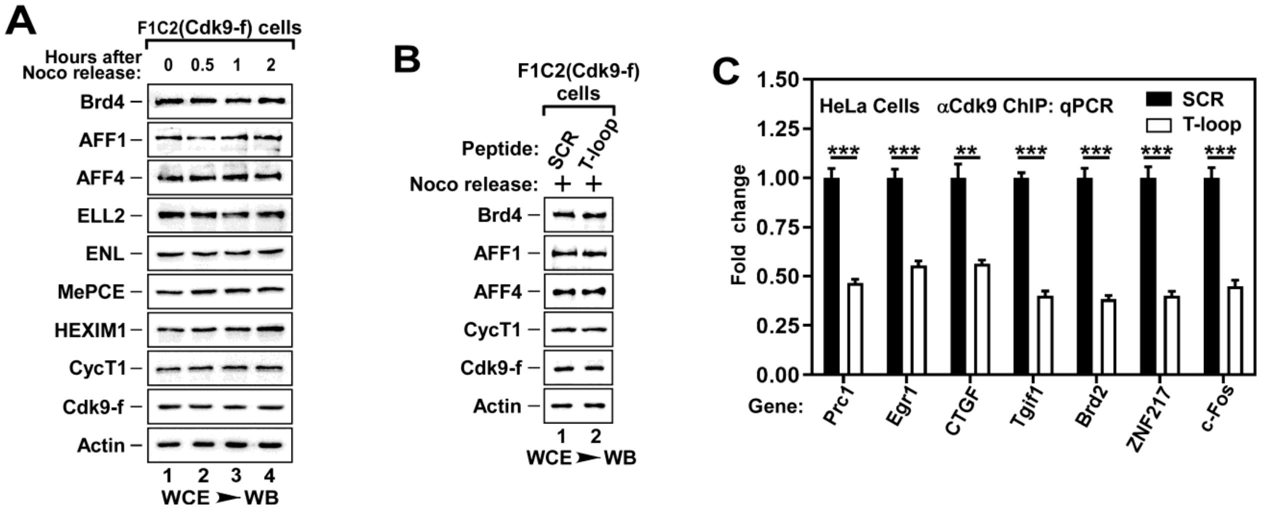

Figure S6. Cdk9 T-loop peptide also blocks Brd4/AFF1/AFF4-mediated reconstitution of active P-TEFb during the cell cycle. (**A**) WB analysis of the expression levels of indicated proteins in F1C2 WCE after Noco release at the indicated time points. (**B**) WB analysis of the expression levels of indicated proteins in F1C2 WCE after Noco release for 2 h. (**C**) ChIP analysis of the levels of Cdk9 on the promoters of indicated G1 genes in Noco-released HeLa cells containing the SCR or T-loop peptides. The values for cells treated with SCR peptide was set to 1.00. **P < 0.01, ***P < 0.001; P-values were assessed using two-tailed Student's t-test.

Supplementary Materials and Methods

Chemicals

Hexamethylene bisacetamide (HMBA, 224235-50G), PP1α (14-595) and PP2B (539568) are from Sigma. Flavopiridol (10009197) is from Cayman Chemical. Okadaic acid (ab141831) is from Abcam. Trizol (15596-026) is from Invitrogen. EDTA-free complete proteinase inhibitor cocktail (4693132001) is from Roche. Reverse transcriptase M-MLV Kit (D2639A) is from Takara Biotech (Dalian, China). Hieff® qPCR SYBR Green Master Mix (11201ES08) is from Yeasen (Shanghai, China). All other chemicals are from Amresco or Sigma.

Antibodies

Rabbit anti-AF4 (AFF1, A302-344A), anti-AFF4 (MCEF, A302-539A), ELL2 (A302-505A), MLLT3 (AF9, A300-596A), ENL (A302-268A) antibodies are from Bethyl Laboratory. Mouse anti-β-Actin antibody (A1978), anti-HA agarose beads (A2095) and anti-Flag M2 affinity resin (A2220) are from Sigma. Rat anti-HA antibody (for WB, 11867423001) is from Roche. Rabbit anti-CDK9 T186 (2549s) is from Cell Signaling Tech. Rabbit anti-Brd4, CDK9, CycT1, Larp7, MePCE and HEXIM1 antibodies were reported previously (1-3).

DNA constructs

The ORF fragments of human Brd4 (NM_001379291.1), AFF1 (NM_005935) and AFF4 (NM_014423) were inserted into a modified pLV-EF1α lentiviral vector and described previously (4). The wild-type CDK9 and its mutations (S175A, S175D, T186A and T186E) were previously reported (1). The S175D/T186E and K48R/E66A mutations of CDK9 were generated by the COP-QuickChange (COP-QC) protocol described previously (5). The truncated Brd4 mutants (i.e., 1-720, 721-1362, 721-1324, 721-1100 and 721-900), AFF1 mutants (i.e., 1-328 and 721-900) and AFF4 mutants (i.e., 1-300 and 301-1163) were generated by PCR. All mutants were subcloned into pRK5 vector, and verified by sequencing. The short hairpin RNAs (shRNAs) targeting human Brd4, AFF1, AFF4, ENL, AF9, ELL2, MePCE and PP1α mRNA were described previously (1,3,4,6). The sequences of primer used for qPCR, ChIP-qPCR, COP-QC and cDNA cloning in this study are as following:

Primer sequences used for qPCR:

Prc1-F	GCGTGAGGAGTTTGTCAGTAT
Prc2-R	GGAGCAGACTTTCTGTGTAGTC
Egr1-F	GCGTGAGGAGTTTGTCAGTAT
Egr1-R	GGAGCAGACTTTCTGTGTAGTC
CTGF-F	GCCCAGACCCAACTATGATTAG
CTGF-R	TCTCCGTACATCTTCCTGTAGT
Tgif1-F	GACATTCCCTTGGACCTTTCT
Tgif1-R	GACCACTCTGTGTATTCGTACTT
Brd2-F	GAGGAGGAAGAAGAGAGTGAAAG
Brd2-R	CGTAGGCAGGAAAGGACATAG
ZNF217-F	CAGGAAACATGCCAACTCAATC
ZNF217-R	CCCACATACCTCACAGCTAAAT
c-FOS-F	CTCAGTGGAACCTGTCAAGAG
c-FOS-R	AAGACGAAGGAAGACGTGTAAG

Primer sequences used for ChIP-qPCR:

c-Fos-P-F	TGAGCCCGTGACGTTTAC
c-Fos-P-R	TGCAGATGCGGTTGGAG
Egr1-P-F	GACCCGTTCGGATCCTTTC
Egr1-P-R	TGCTCAGCAGCATCATCTC
ZNF217-P-F	GGAATCCGGGAGGTGTC
ZNF217-P-R	GGGAACTGAGGTCATCCTG
Tgif1-P-F	TCTGTGTCTTCCTCCATCCA
Tgif1-P-R	GTTGTCCTGGAGGTGTCTTTC
CTGF-P-F	GAGTGGTGCGAAGAGGATAG
CTGF-P-R	TCCTACACAAACAGGGACATT
Brd2-P-F	CTCTCTCCAGACCCTCATATCT
Brd2-P-R	CGAAACCCAGAGCGCTATAA
Prc1-P-F	AGGAGTCCCTTGAGGCT
Prc1-P-R	TCCAGGTCCAGACCTACTC

Primer sequences used for COP-QC mutagenesis:

Cdk9 T186E-F	AGCCAGCCCAACCGCTACGAAAACCGTGTGG
Cdk9 T186E-R	CAGAGTGTCACCACACGGTTTTCGTAGCGGTTG
Cdk9 K48R -F	GGTGGCTCTG AGG AAGGTGCTGATGGAAAAC
Cdk9 K48R -R	ATCAGCACCTT CCT CAGAGCCACCTTCTGG
Cdk9 E66A-F	CCCCATTACAGCCTTGCGGGCGATCAAGATCCTT
Cdk9 E66A-R	GTGTTTTAGAAGCTGAAGGATCTTGATCGCCCGCAAGGC

Primer sequences used for cDNA cloning:

Brd4 1-720-F	CGGGAATTCCCGGGATCCATGTCTGCGGAGAGCGGC
Brd4 1-720-R	GTAGTCAAGCTTTCTAGACTCTGTTTCGGAGTCTTCGCTGT
Brd4 721-1362-F	CGGGAATTCCCGGGATCCATGGCTCCGAAGTCAAAAAGAAGGG
Brd4 721-1362-R	GTAGTCAAGCTTTCTAGAGAAAAGATTTTCTTCAAATATTGACAATAGAT CAC
AFF1 1-328-F	CGGGAATTCCCGGGATCCATGGCAGCCCAGTCAAGTTTGT
AFF1 1-328-R	GTAGTCAAGCTTTCTAGATTTCAAGTCTGTTTTTCAAAGGTCTG
AFF1 329-1210-F	CGGGAATTCCCGGGATCCGTGCCTGCCAAAGCCAAGC
AFF1 329-1210-R	GTAGTCAAGCTTTCTAGAAGGTGTTTTGGTTAATTCTTGTAGCTG
AFF4 1-300-F	CGGGAATTCCCGGGATCCATGAACCGTGAAGACCGGAATGT
AFF4 1-300-R	GTAGTCAAGCTTTCTAGATATTTTCAGCTTGGTGAGATGTGCTTT
AFF4 300-1163-F	CGGGAATTCCCGGGATCCCCTTCCCAACCACTGGATGCAT
AFF4 300-1163-R	GTAGTCAAGCTTTCTAGAAGATATCAACTTGGCATCCTGGC

Preparation of LSF, HSF and WCE

The low-salt fraction (LSF) and high-salt fraction (HSF) were prepared with improved nuclear fractionation protocol. Briefly, HeLa cells were collected with recording the packed cell volume (PCV), and swollen in 5×PCV of ice-cold Buffer A as previously described (7,8). After centrifuged, the cell pellets were resuspended and extracted in equal volume of low-salt Buffer (10 mM HEPES pH 7.9/1.5 mM $MgCl_2$/150 mM KCl/1 % NP-40/1 mM DTT/0.5 mM PMSF/1×protease inhibitor cocktail) on ice for 20 min followed by centrifugation at 5000×g, 4^oC for 2 min. The supernatant was saved as the low-salt fraction (LSF, ~3.6×PCV, containing chromatin-free P-TEFb). For the preparation of high-salt fraction (HSF, containing chromatin-bound P-TEFb), the

low-salt extracted nuclei (LSEN) were extracted with 3.6×PCV of high-salt buffer (10 mM HEPES pH 7.9/20% Glycerol/0.3 M NaCl/1.5 mM $MgCl_2$/0.4 mM EDTA/0.5% NP40/1 mM DTT/ 0.5 mM PMSF/1×protease inhibitor cocktail) on a rotator at 4°C for 30 min and centrifuged at 12,000×g at 4°C for 10 min. The supernatant was saved as the HSF. Finally, the salt concentration of LSF was brought to the same as those of HSF before immunoprecipitation. For the preparation of whole cell extraction (WCE), the Buffer A-swollen cells were subjected to equal volume of high-salt buffer with 0.6 M NaCl on a rotator at 4°C for 30 min. The nuclei were centrifuged at 12 000g at 4C for 10 min and the supernatant was saved as WCE. The LSF, HSF and WCE were stored in -80°C, or used directly.

Supplementary References

1. Chen, R., Liu, M., Li, H., Xue, Y., Ramey, W.N., He, N., Ai, N., Luo, H., Zhu, Y., Zhou, N. *et al.* (2008) PP2B and PP1alpha cooperatively disrupt 7SK snRNP to release P-TEFb for transcription in response to Ca2+ signaling. *Genes Dev*, **22**, 1356-1368.
2. He, N., Jahchan, N.S., Hong, E., Li, Q., Bayfield, M.A., Maraia, R.J., Luo, K. and Zhou, Q. (2008) A La-related protein modulates 7SK snRNP integrity to suppress P-TEFb-dependent transcriptional elongation and tumorigenesis. *Mol Cell*, **29**, 588-599.
3. Xue, Y., Yang, Z., Chen, R. and Zhou, Q. (2010) A capping-independent function of MePCE in stabilizing 7SK snRNA and facilitating the assembly of 7SK snRNP. *Nucleic Acids Res*, **38**, 360-369.
4. Lu, X., Zhu, X., Li, Y., Liu, M., Yu, B., Wang, Y., Rao, M., Yang, H., Zhou, K., Wang, Y. *et al.* (2016) Multiple P-TEFbs cooperatively regulate the release of promoter-proximally paused RNA polymerase II. *Nucleic Acids Res*, **44**, 6853-6867.
5. Wang, H., Zhou, N., Ding, F., Li, Z., Chen, R., Han, A. and Liu, R. (2011) An efficient approach for site-directed mutagenesis using central overlapping primers. *Anal Biochem*, **418**, 304-306.
6. He, N., Liu, M., Hsu, J., Xue, Y., Chou, S., Burlingame, A., Krogan, N.J., Alber, T. and Zhou, Q. (2010) HIV-1 Tat and host AFF4 recruit two transcription elongation factors into a bifunctional complex for coordinated activation of HIV-1 transcription. *Mol Cell*, **38**, 428-438.
7. Ai, N., Hu, X., Ding, F., Yu, B., Wang, H., Lu, X., Zhang, K., Li, Y., Han, A., Lin, W. *et al.* (2011) Signal-induced Brd4 release from chromatin is essential for its role transition from chromatin targeting to transcriptional regulation. *Nucleic Acids Res*, **39**, 9592-9604.
8. Hu, X., Lu, X., Liu, R., Ai, N., Cao, Z., Li, Y., Liu, J., Yu, B., Liu, K., Wang, H. *et al.* (2014) Histone cross-talk connects protein phosphatase 1alpha (PP1alpha) and histone deacetylase (HDAC) pathways to regulate the functional transition of bromodomain-containing 4 (BRD4) for inducible gene expression. *J Biol Chem*, **289**, 23154-23167.
